# An integrated surgical training program for hepatic cystic echinococcosis in Xinjiang of China

**DOI:** 10.1371/journal.pntd.0008023

**Published:** 2020-03-12

**Authors:** Hongwei Zhang, Jian Yang, Jiang Li, Jing Yang, Yunbao Yu, Guisheng Liu, Yongguo Zhang, Long Zhang, Wei Guo, Abuduwayiti, Hong Sun, Shuxia Guo, Xueling Chen, Xiangwei Wu, Shijie Zhang, Xinyu Peng

**Affiliations:** 1 Department of hepatobiliary surgery, the First Affiliated Hospital, School of Medicine, Shihezi University, Xinjiang, China; 2 Tongji Medical School, Huazhong University of Science and Technology, Wuhan, Hubei, China; 3 Department of Preventive, School of Medicine, Shihezi University, Xinjiang, China; 4 Department of Immunology, School of Medicine, Shihezi University, Xinjiang, China; IRNASA, CSIC, SPAIN

## Abstract

**Background:**

Human cystic echinococcosis (CE) is one of the commonest zoonoses, and it is endemic in many parts of the world including China. Complications and recurrences after the surgical treatment of hepatic CE (HCE) incur a large personal, healthcare, and societal burden. There has been some progress in HCE prevention, diagnosis, and treatment, but there is no “one size fits all” approach, and surgery still remains the cornerstone of treatment for some cyst stages and locations or in areas with little knowledge or access to other treatment modalities. In 2009 we designed and implemented a program to improve surgical outcomes from HCE in Xinjiang province, China.

**Methodology/Principal findings:**

A multimodal HCE training program was implemented in eleven primary hospitals in Xinjiang province, China, which provided education and training on HCE clinical knowledge and practice, the application of diagnostic and treatment options, and optimal surgery. The management of HCE cases was analyzed before and after program implementation. Contrast enhanced CT use, application of scoloicidal agents, removal of necrotic cyst wall remnants, appropriate perioperative drug use, and the use of optimal surgical approach increased after program implementation. Further, postoperative recurrences and residual cavity complications creased from 7.4% to 1.3% and 15.2% to 9.0% after program implementation, respectively.

**Conclusions/Significance:**

Tis integrated surgical training program is useful for improving outcomes of patients with HCE and can be used in institutions in other endemic areas.

## Author summary

Hepatic cystic echinococcosis (HCE) is an endemic and neglected global disease. Many deprived areas in China still lack the knowledge and expertise to successfully treat HCE surgically, which results in high postoperative complication and recurrence rates that further decrease postoperative quality of life. In this study, we aimed to transfer our extensive experience in the development and implementation of effective surgery for HCE to eleven hospitals in the *Echinococcus granulosu*s endemic province of Xinjiang to improve the surgical treatment of HCE. Our results indicate that our training program effectively reduces recurrence and complication rates and will be of use in other endemic areas.

## Introduction

Human cystic echinococcosis (CE) is a common zoonosis caused by the larval stage of *Echinococcus granulosu*s, which progresses mainly as liver and lung cysts [1]. Human echinococcosis remains a significant medical and economic burden in endemic areas globally [2]. The disease is neglected at least in part because it tends to affect underdeveloped rural areas, including in western China. Given its impact on human health, the scientific community and public health stakeholders have allocated significant resources to combatting this parasitic disease [3]

At present, echinococcosis control in western China mainly focuses on the preventative measure of deworming dogs with praziquantel; however, this approach has proven difficult in local semi-nomadic communities [4]. HCE can, therefore, be regarded as a failure of primary prevention policies, in turn conferring significant economic and physical burdens on patients, healthcare infrastructures, and society. Surgery remains the only radical treatment for cystic echinococcosis, but postoperative recurrence (3.2–22%) and residual cavity complications (10–47%) remain common [5–13]. This is especially true in Xinjiang Province.

In 2005, the Chinese government launched a major echinococcosis control program in 217 counties in western China. This program, organized by the National Health and Family Planning Commission (NHFPC, formerly the Ministry of Health) of the People's Republic of China and the “Central subsidies for local free treatment of hydatid disease” project, relieved patients of the financial burden of CE treatment. Our previous pilot study of eleven primary hospitals in Xinjiang province, a typical endemic province in western China, showed that the majority of HCE patients were treated with traditional endocystectomy in primary hospitals [14]. However, recurrences and postoperative complications (residual cavity infection, biliary fistula) were common after surgery. Further, there was a lack of basic knowledge on CE and surgical treatment skills in healthcare providers, and perioperative medication management was very poor.

We previously developed a radical surgical procedure, sub-adventitial pericystectomy, which, together with perioperative techniques (CT cyst evaluation; management of the surrounding ducts and residual cavity; application of scolecidal agents), significantly decreased recurrences and postoperative complications [15]. This radical procedure, later renamed sub-adventitial cystectomy, consisted of cyst resection along a true and less bloody plane between the cyst adventitia and healthy hepatic tissue, and was adopted by the WHO Informal Working Group on Echinococcosis (WHO-IWGE) and some experts as a safe surgical option [16,17]. As a result, we targeted another eleven medical centers in endemic areas to improve outcomes for patients with HCE.

Here we report outcomes from this program to improve the effectiveness of HCE surgery in underdeveloped areas in Xinjiang. Our approach represents an integrated surgical protocol including HCE diagnostic and treatment knowledge, surgery, and related auxiliary technologies (CT evaluation of the cysts and surrounding ducts; the application of an optimal scolecidal agent; management of the encountered bile ducts, vessels, and remnant wall; full protection of the surgical field). We report encouraging results after program implementation which are likely to improve the surgical management of HCE globally.

## Methods

### The research area

Eleven cities in echinococcosis endemic areas in Xinjiang were selected as the study fields, and eleven hospitals localized in these cities were chosen as demonstration units. Most target hospitals were primary level 2 hospitals (except one level 3 hospital): Xinjiang Production and Construction Corps Hospital (level 3); Xinjiang Production and Construction Corps Fourth Division Hospital; Fifth Division Hospital; Sixth Division Hospital; Ninth Division Hospital; Tenth Division Hospital; Thirteenth Division Hospital; Hami Prefecture Central Hospital; Altay Region People's Hospital; Usu City People's Hospital; and Shawan County People's Hospital.

### Program structure

The program was conducted in three stages. The first was a baseline survey of knowledge and current practice, which included an HCE knowledge survey for health care personnel and the retrospective review of selected surgical cases over the preceding three years (follow-up duration 36 months). In this stage, the surgical management of each patient was established by reviewing the surgical approach and the preoperative, intraoperative, and postoperative treatment measures by retrospective case review of the medical records, especially surgical records.

Surgical complications assessed were residual cavity complications: bile leakage, detected by biochemical testing of the drain; and residual cavity infection, evaluated according to symptom occurrence such as fever and/or chills and bacterial culture of drain fluid. Patients were followed by ultrasonography at 3, 6, 12, 24, and 36 months [18]. Recurrences were defined as re-formation of the cyst in the same place and/or in the abdomen after the primary operation during follow up period.

Two to three surgical state-appointed experts evaluated the surgical techniques of the main operators and recorded the cyst classification, surgical methods, residual cavity management, and scolecidal agent use. A 12-item questionnaire was administered to health care personnel before and after training to determine their knowledge about echinococcosis, where each question was scored correct (knew the answer) or incorrect (did not know the answer) (**S1 Text**).

The second stage of the program was training over six months. Based on the results of the health care personnel survey, we compiled lectures, video training materials, built a remote teaching network to assist in diagnosis and treatment, and organized training courses for health care personnel to learn about pre-operative CT evaluation (**S2 Text**), indications for surgery, our radical surgical procedure and its derivative techniques (such as how to find the space between the cyst and healthy hepatic tissue; how to differentiate functional and non-functional bile ducts and vessels neighboring the cyst, etc.), and the perioperative medical management of HCE. During this period, surgical experts visited each hospital to demonstrate the surgery to further improve the effect of training. Problems encountered during training were communicated with the main operator in a timely manner to make improvements (**S3 Text**).

The third stage of the program was re-evaluation. The knowledge and surgical ability of health care personnel were re-evaluated, and new surgically-treated HCE cases were assessed after the sixth month training period. Surgical personnel were assessed by the three designated experts to ensure that all had acquired the competency to perform sub-adventitial cystectomy and derived surgeries (subtotal excision, partial excision, residual omentoplasty, residual cavity drainage, etc.). New surgically-treated HCE cases were collected and analyzed between 2010 and 2012 with a 36-month duration of follow-up.

### Classification of hydatid cysts

The international classification of ultrasound images in cystic echinococcosis was used: CL: origin uncertain, not given a designation of a CE type lesion; CE 1 and 2: cyst types active, usually fertile and containing viable protoscoleces; CE 3: cysts entering a transitional stage where the integrity of the cyst has been compromised either by the host or by medical treatment. CE 4 and 5: cysts inactive and have normally lost their fertility and are degenerative [16].

### Statistics

Means with standard deviations were used to describe normally distributed data. Student’s t-test was used to compare two groups. Categorical data were evaluated by the chi-square test. A probability value less than 5% was considered significant. R×C chi-square test was used to evaluate the overall difference of the operation proportions.

### Ethics Statement

The ethical committee of the First Affiliated Hospital, School of Medicine, Shihezi University approved the study protocol and all data analyzed were anonymized.

## Results

### Basic demographic information

Six-hundred and forty-four patients were studied prior to program implementation. The average patient age was 43.2±15.5 years and 338 were male and 306 female. The average cyst diameter was 9.6±5.4 cm.

Six-hundred and forty three patients were studied after program implementation, and there were no significant differences in age, gender, cyst location, or cyst type between the pre- and post-program periods (**Table 1**).

**Table 1:** Demographics of the patients before and after study implementation.

Group		Before implementation	After implementation	t/χ^2^	p-value
Age(year)		43.2±15.5	41.8±14.7	1.602	0.109
Gender	Male	338	314	1.716	0.190
	Female	306	329	3.79	0.15
Cyst location	R	299	265		
	L	93	108		
	B	252	270		
Total		644	643		
Cyst type	CE1	32	44	9.41	0.094
	CE2	358	382		
	CE3a	18	24		
	CE3b	213	180		
	CE4	10	7		
	CE5	13	6		
Total		644	643		
Cyst diameter (cm)		9.6±5.4	10.2±5.4	-1.765	0.078

Abbreviations: R: right lobe; L: left lobe; B: both lobes.

### Possible influencing factors determined by baseline investigation

After baseline information investigation of the eleven study hospitals and 644 surgically-treated HCE cases, possible factors influencing diagnosis and outcomes were: (1) low HEC knowledge of the health care personnel; (2) low CT imaging use; (3) the most effective protoscolex-inactivating agents were not used intraoperatively; (4) few surgical procedures were applied to manage the fibrotic and necrotic tissues of the residual cyst wall during open surgery; (5) perioperative medical management was poor; (6) traditional endocystectomy and its derivatives were the main surgical procedures used to treat HCE.

### The HCE knowledge level of health care personnel was improved

**Fig 1:**
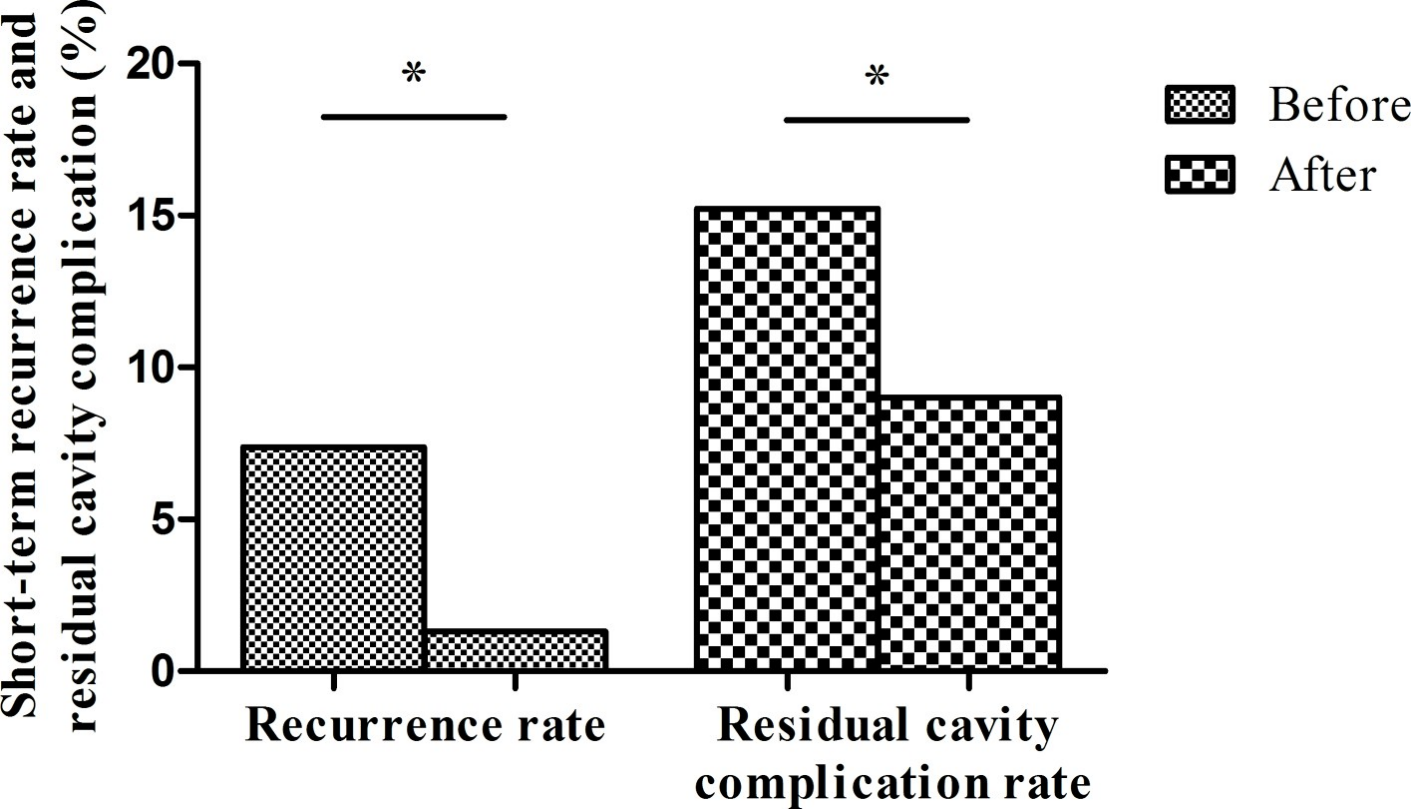
HCE knowledge of health care personnel before and after program implementation. Before program implementation, HCE knowledge of most of health care personnel was poor. After program implementation, knowledge increased. *p<0.05 by the chi-square test. 1–12 denote the following questions: 1. Characteristics of echinococcosis; 2. The definitive host and intermediate host of cystic echinococcosis; 3. Most affected organs; 4. Drug of first choice; 5. Best radical surgery; 6. Intraoperative scolecidal agents and acting time; 7. Precautions in radical surgical approaches; 8. Sub-adventitial cystectomy in the surgical treatment of HCE; 9. Contrast enhanced CT application in HCE diagnosis and management; 10. Immunological diagnostic methods; 11. Processing methods of slaughtered livestock with hydatid cysts; 12. Hydatid specimen collection and storage.

The HCE knowledge of 549 personnel managing HCE cases (surgeons, nurses, lab staff, radiologists) in the 11 hospitals was investigated. At baseline, knowledge of hydatid disease was generally poorly understood by health care personnel, suggesting that training and education were needed. After program implementation, 533 health care personnel were investigated. The level of all knowledge about CE significantly improved (p<0.05; **Fig 1**).

### Program implementation increased the use of CT imaging

**Fig 2:**
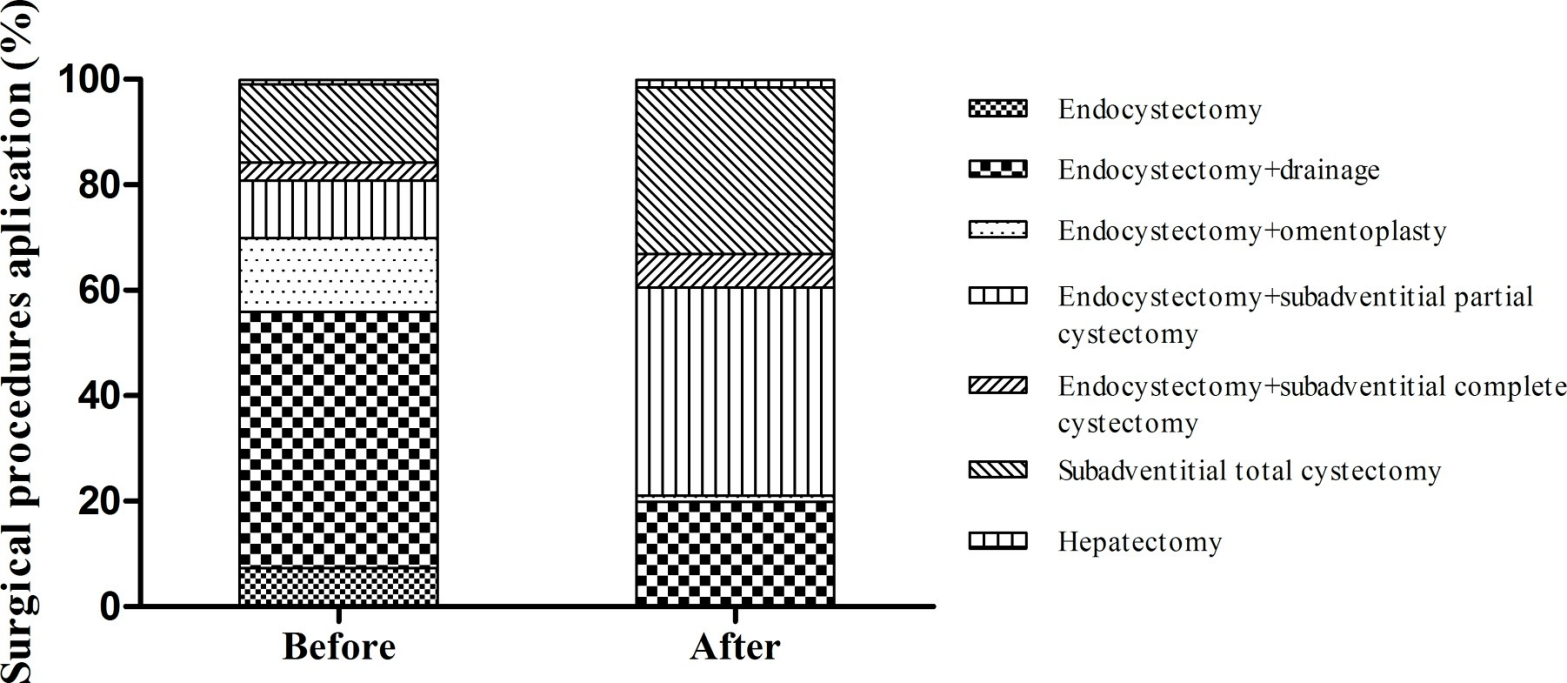
Contrast enhanced CT use before and after program implementation. Before program implementation, most CT examinations were plain scans. After program implementation, the use of contrast enhanced CT scans increased to 91.14%. *p<0.001 by the chi-square test.

Ultrasound remains the cornerstone of HCE diagnosis, staging, and follow-up [19]. However, in our experience, contrast-contrast enhanced CT imaging has added value, being objective, accurate, and better defining the relationship between the hydatid cyst and surrounding vessels (bile ducts). 91.3% of patients received ultrasound and 70.7% (455/644) of patients received CT imaging prior to the operation, most of which were plain scans (**Fig 2**). The program trained health care personnel to read CT images before and during the operation and implemented a remote medical consultation system between hospitals, departments, and personnel to assist in CT interpretation. After program implementation, the use of contrast enhanced CT imaging significantly increased to 91.1% (586/643; χ^2^ = 87.31, p<0.001) (**Fig 2**).

### Use of effective protoscolex-inactivating agents before and after program implementation

**Fig 3:**
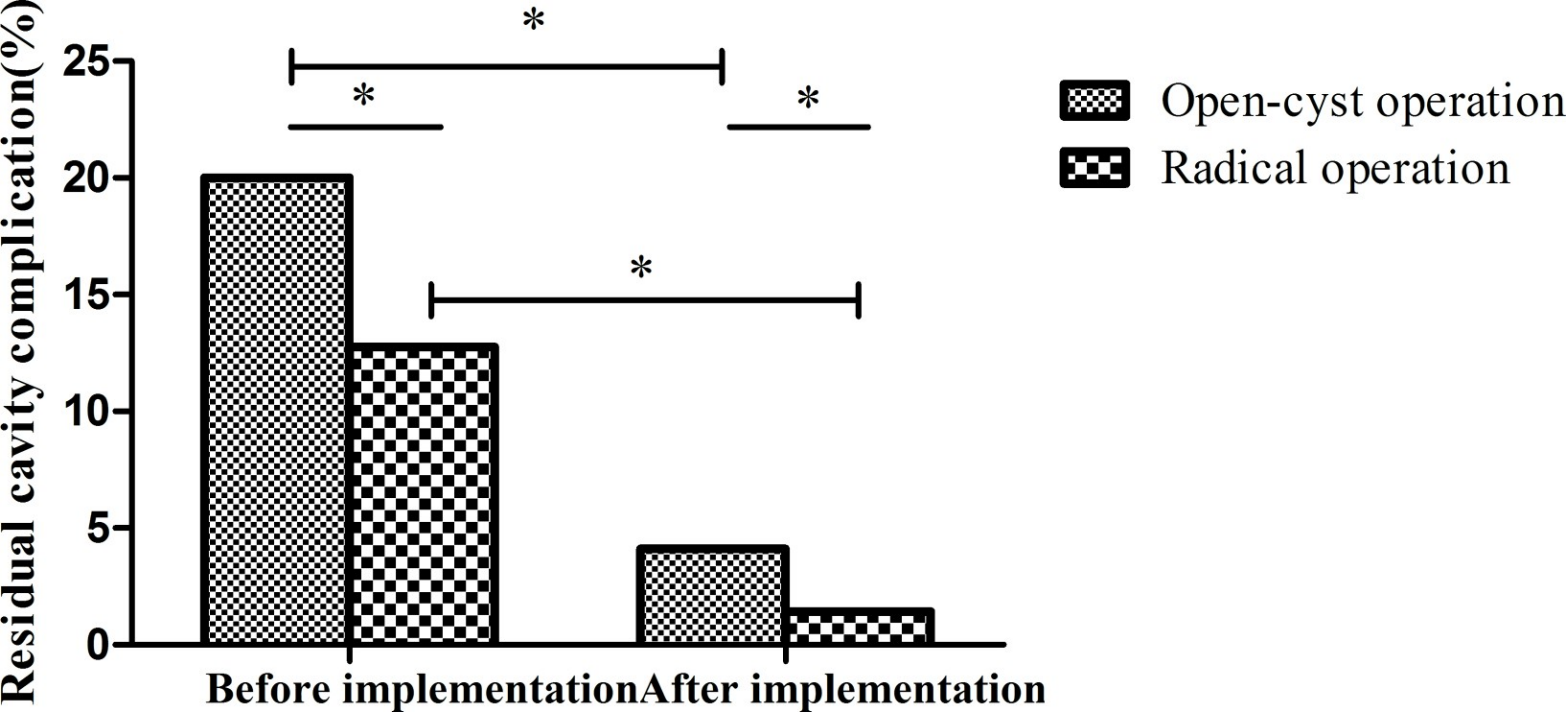
Use of scolecidal agents in open cyst operations before and after program implementation. Before program implementation, 3% hydrogen peroxide was used in 56.6% of cases and hypertonic saline in 35.4% of cases. After program implementation, hypertonic saline used increased to 82.3% and the application of 10% formalin decreased to 0%. *p<0.001 by the chi-square test.

Intraoperative hydatid scolex leakage is an important cause of postoperative recurrence. Therefore, the effective use of scolecidal agents is key to reducing recurrence, with the WHO Expert Consensus recommending 20% hypertonic saline. Before program implementation, of 542 patients undergoing endocystectomy, only 35.4% (192/542) used 20% hypertonic saline and 56.6% (307/542) used 3% hydrogen peroxide (**Fig 3**). There was no standardized protocol for scolecidal use in these hospitals. After program implementation, of 430 patients receiving open cyst operations, hypertonic saline use significantly increased to 82.3% (354/430; χ^2^ = 214.24, p<0.001) (**Fig 3**).

### Improved handling of fibrotic or necrotic tissues during open surgery

**Fig 4:**
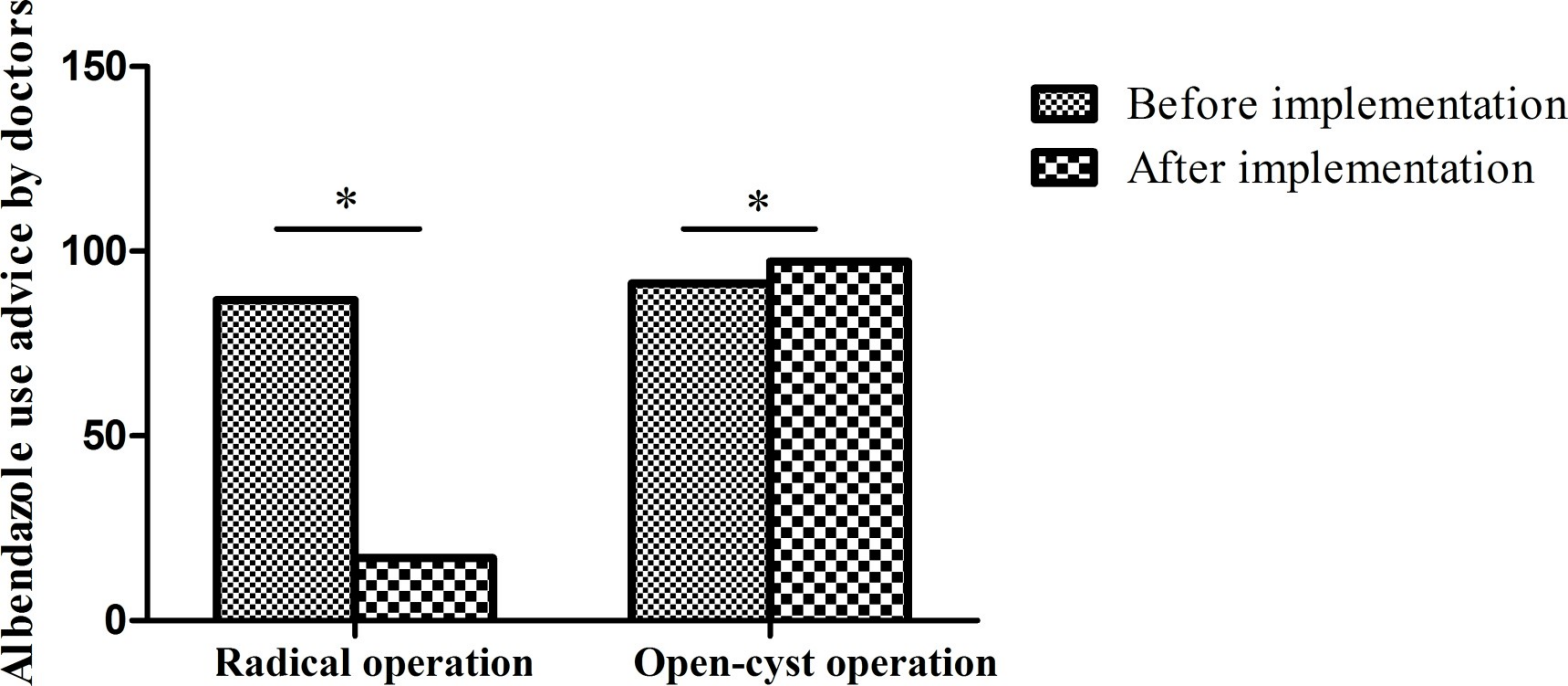
Handling of fibrotic and necrotic tissues during open surgery before and after program implementation. Before program implementation, only 20.1% of surgeries involved removing necrotic and fibrotic tissue. After program, implementation, the proportion increased to 47.9%. *p<0.001 by the chi-square test.

HCE walls can be necrotic, with residual necrotic tissue sometimes causing residual cavity infection or bile leakage. Furthermore, small bile communications may persist in the fibrotic or necrotic wall. According to our experience, the residual cyst wall should be removed and explored during surgery. Maximized removal of the residual cystic and necrotic wall can effectively reduce postoperative residual cavity infection and recurrence rates [15]. For cystic walls that are difficult to remove, the simplest and most effective approach is to eliminate the fibrotic or necrotic tissue by electrotome, which is a simple and suitable technique in any hospital. Prior to program implementation, 109 patients (20.1%, 109/542) had residual cyst wall resections and explorations. Our program provided training on the handling of necrotic and fibrotic tissues, which was demonstrated during live operations. After program implementation, 206 patients (47.9%, 206/430) received residual cyst wall resections with exploration (χ^2^ = 84.57, p<0.001) (**Fig 4**).

### Perioperative standard use of medication improved

**Fig 5:**
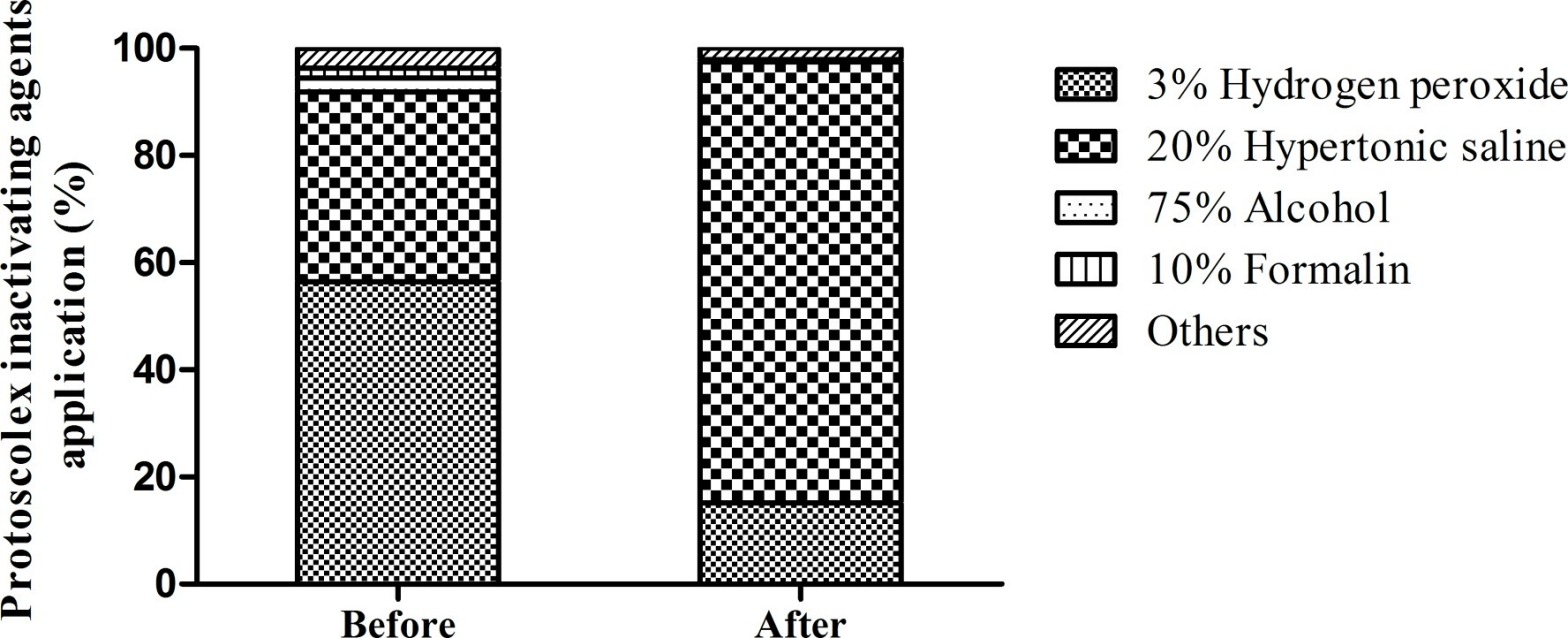
Before program implementation, use of preoperative and postoperative albendazole was low (6.4%). After program implementation, albendazole use increased to 20.5%. (*p<0.001 by the chi-square test).

Many doctors did not prescribe albendazole 7 days before surgery 6.4% (41/644). After program implementation, preoperative medication use increased to 20.53% (132/643; χ^2^ = 55.46, p<0.001) (**Fig 5**).

**Fig 6:**
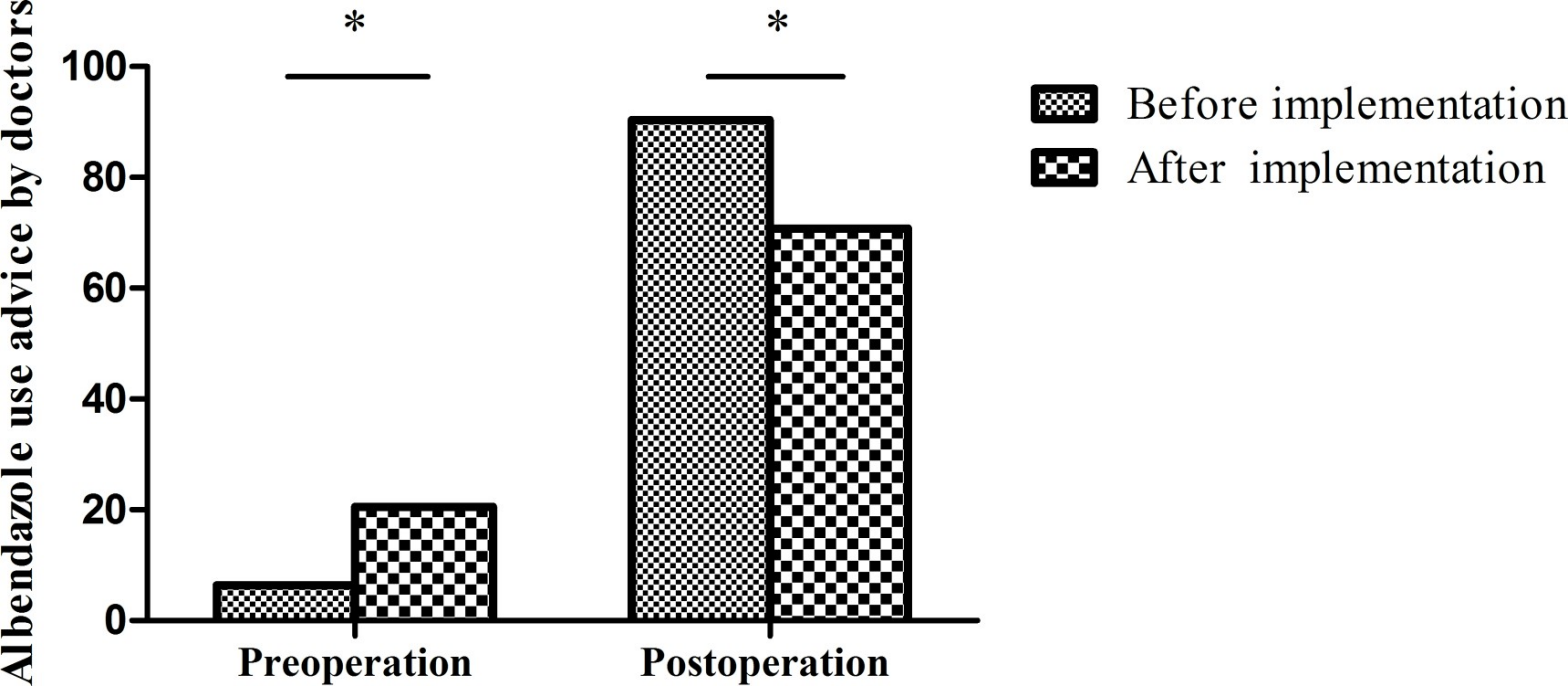
For the doctor's discharge advice, the open-cyst operations with albendazole use increased, while the radical operation with albendazole use significantly decreased. (*p<0.001 by the chi-square test).

Before the implementation, 90.37% (582/644) cases of oral albendazole were mentioned in the discharge guidance of postoperative hydatid patients; after the implementation, 70.76% (455/643) cases of oral albendazole were mentioned in the discharge guidance(χ^2^ = 79.06, p<0.001) (**Fig 5**). Among the patients with open-cyst surgery, 91.3% (411/450) patients were prompted to continue oral albendazole in discharge guidance before the implementation. After the implementation, 97.2% (419/431) patients with open surgery were advised to continue oral albendazole in the discharge guidance (χ^2^ = 13.97, p<0.001). For the radical surgery, before implementation, 88.1% (171/194) were told to continue intake albendazole in discharge guidance, however, 17.0%(36/212) patients were told to continue with albendazole after implementation (χ^2^ = 199.15, p<0.001). It showed that the use of albendazole was more rational after the implementation(**Fig 6**).

### The use of traditional endocystectomy and its derivatives decreased

**Fig 7:**
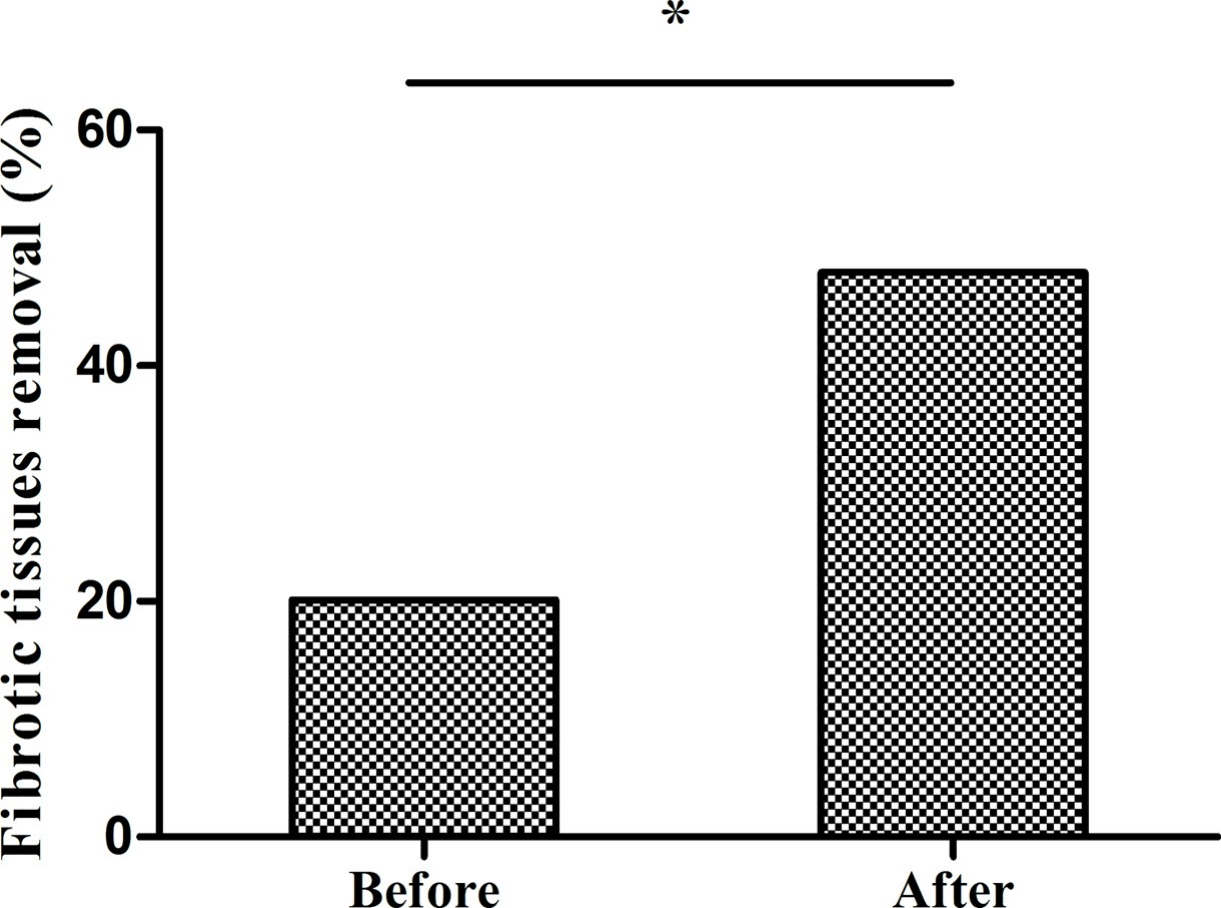
The proportion of patients undergoing radical surgery improved. Endocystectomy and its derivatives (drainage; omentoplasty) decreased after program implementation; especially the sole use of endocystectomy (0%). Radical surgery, especially subadventitial (total and complete) cystectomy, increased. *p<0.001 by the chi-square test.

A variety of surgical procedures are used to manage HCE, with endocystectomy a traditional method used in the primary hospitals of Xinjiang province. Before the program, 70% (450/644) of patients received endocystectomy and its derivatives (the opening cyst cavity open; the opening cyst cavity drainage; the opening cyst cavity omentoplasty), with pericystectomy performed in 29.2% (188/644) of patients. Radical surgery was only undertaken in 15.8% (102/644) of cases, 88.2% (90/102) of which were sub-adventitial cystectomies (**Fig 7**).

After this program, the proportions of the surgical procedures were changed. No more simple “endocystectomy” surgery without residual cavity drainage and omentoplasty (decreased from7.5% to 0%) was performed. And 21.0% (135/643) cases were performed by “endocystectomy” and its derivatives; 45.9% (295/643) received cystectomy. Radical surgery took 32.9% (212/643). Compared with that before of the program, overall proportion change of all surgical procedures was statistical significant (χ^2^ = 216.56, P < 0.001) **(Fig 7)**.

### The overall mortality rate was zero and recurrence and postoperative complications decreased

**Fig 8:**
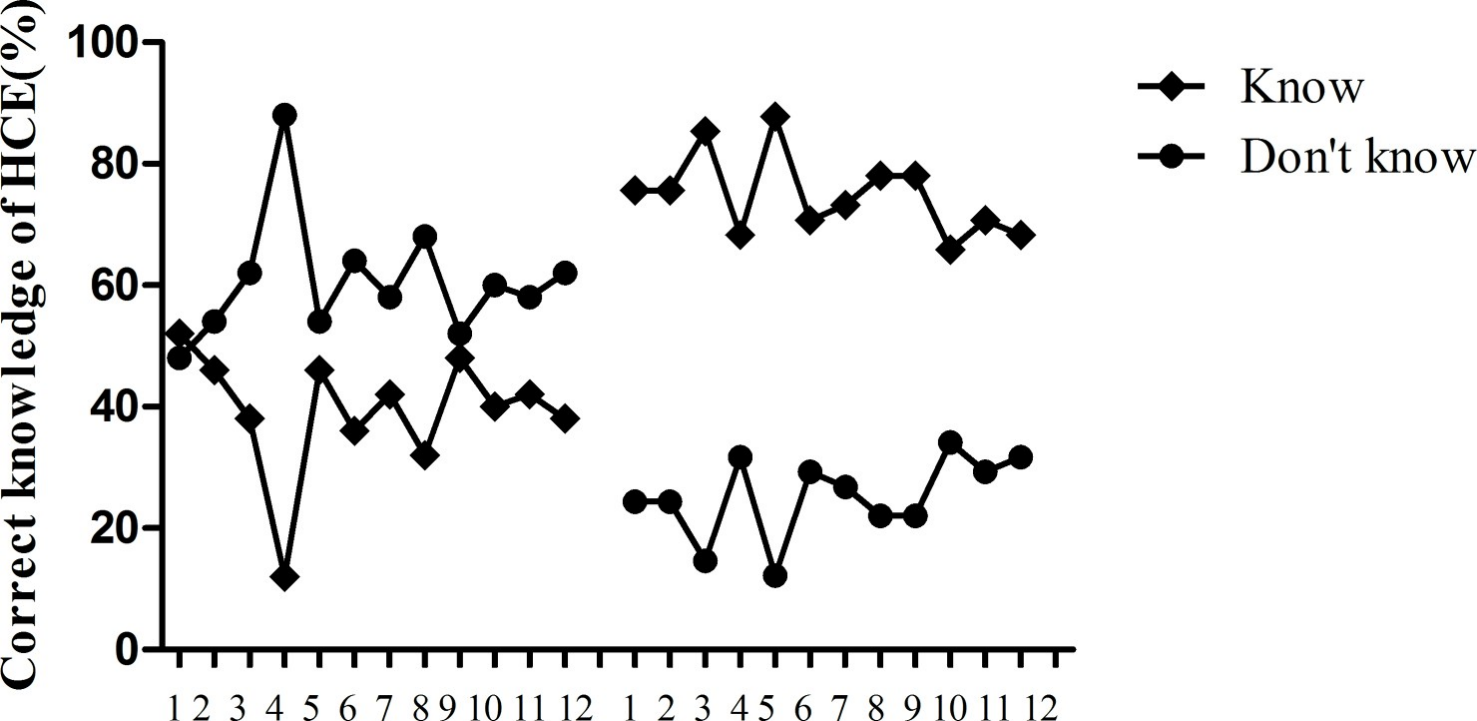
Decreases in short-term overall postoperative recurrences and residual cavity complications after program implementation. Recurrences decreased from 7.4% to 1.3%, while residual cavity complications decreased from 15.2% to 9.0%. *p<0.001 by the chi-square test.

Prior to program implementation, postoperative residual cavity complications occurred in 15.2% (98/644) cases and recurrences in 7.4% (**Fig 8**). After program implementation, postoperative residual cavity complications decreased to 9.0% (58/643; χ^2^ = 13.48, p<0.01) and recurrences decreased to 1.3% (χ^2^ = 11.6, p<0.001) (**Fig 8**). The mortality rate of these operations was zero.

**Fig 9:**
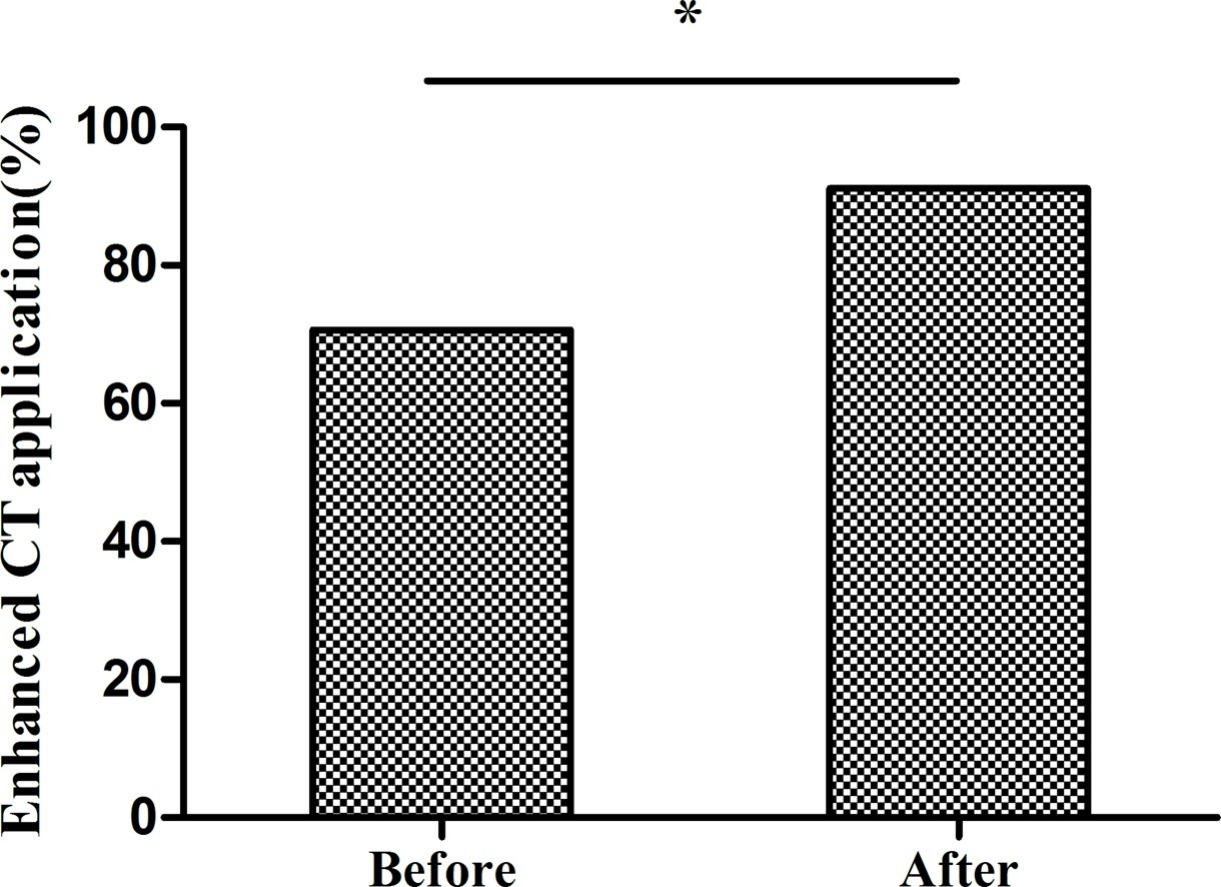
After the implementation, the residual cavity complications of the open- and closed-cyst operation decreased compared with that before the demonstration, and the difference was statistically significant. *p<0.001 by the chi-square test.

We analyzed the incidence of residual cavity complications in open- and closed- cyst surgery. The results showed that the complication rate of open cyst operation before implementation was 20% (90/450), while that of closed operation was 4.12% (8/194), the difference was statistically significant(χ^2^ = 26.48, p<0.001). The complication rate of open operation after demonstration was 12.76%(55/431), while that of closed operation was 1.42% (3/212), the difference was statistically significant(χ^2^ = 22.29, p<0.001). This indicated that the complication rate of open operation was higher than that of closed operation. We further analyzed the difference in the complication rate between the open operation and the closed operation before and after the demonstration, and the results indicated that the difference in the open operation was statistically significant(χ^2^ = 8.39, p = 0.004), while the difference in the closed operation was not, indicating that the residual cavity complication rate of the open operation after the demonstration was significantly lower than that before the demonstration(χ^2^ = 2.82, p = 0.093) (**Fig 9**).

## Discussion

HCE is a disease that disproportionately affects the pasturing areas and half-pasturing areas, most of these places are impoverished rural areas in China. It is widely accepted that the management of echinococcosis may be medical or surgical and should be planned in an individualized manner [16]. According to the IWGE-advocated stage-specific approach, “watch and wait” is appropriate for patients with CE4-CE5 stage cyst; patients CE2 and CE3b stage cyst can be managed surgically or percutaneously; while surgery is not recommended for patients with CE1 and CE3a cysts, who are best managed with percutaneous treatment and drugs. Surgical procedures, including radical (cystectomy and hepatic resection) and conservative (deroofing and tube drainage, omentoplasty, etc.) approaches, were originally considered to be the first choice for HCE [20, 21]. However, the high probability of postoperative complications and recurrences remains a major problem affecting quality of life an incurring further economic burden [22].

Xinjiang is located in northwestern China and is large in area. Many areas Xinjiang are pastoral areas. There are cattle, sheep, as well as dogs and wolves and other animals, therefore, these areas are prone to echinococcosis. In addition, the development of these areas is relatively backward and the medical conditions are poor. Local hospitals suffer from poor training opportunities and slow uptake of newer diagnostic and treatment approaches. For HCE, many hospitals and surgeons still use endocystectomy with its consequent postoperative complications and recurrence rates. In order to improve the surgical treatment of HCE in Xinjiang, we integrated our surgical and wider management experience and promoted it in eleven primary hospitals, with good results.

We first conducted a retrospective study of the management of HCE cases in Xinjiang to establish a baseline for the study hospitals and to establish possible adverse factors affecting surgical outcomes. We assessed the facilities available in the hospitals to assess whether they were able to perform radical surgery; all eleven hospitals were able to perform sub-adventitial cystectomy [14]. With respect to prior knowledge of the health care personnel, the main problems likely influencing the effective treatment of HCE were knowledge levels about the condition, preoperative assessment, standard perioperative medication application, surgical procedures, and scolecidal agent application. We integrated the latest consensus expertise in surgical management and our own experience into an established HCE surgical treatment protocol, which was then promoted in the primary hospitals, as described previously [15]. Our results show that the training administered in this program was effective and resulted in improvements in HCE knowledge and perioperative skills.

Surgical treatment for HCE can be classified into two main categories: radical and non-radical. In general, radical surgery results in fewer recurrences and postoperative complications [23]. We adopted sub-adventitial (total and partial) cystectomy as the radical surgery due to its extremely low recurrence rates in elective patients in our hospital. As important as the main operation, the auxiliary techniques (surgical field protection; management of the remnant cyst wall and functional and non-functional bile ducts and vessels; the application of preoperative CT evaluation, perioperative medication, etc.) require attention during open cyst surgeries. It is now established that the opening of the cyst is the main reason for recurrence; however, our integrated program successfully decreased recurrences from 7.4% to 1.3% for combined the open and closed cyst procedures.

With respect to postoperative complications, remnant tissues in the residual cavity are the main reason for postoperative residual cavity complications [24]. Further, biliary fistulas can be concealed within the remaining outer capsule wall, which can lead to bile leakage, a common postoperative complication with variable incidence [25–27]. In our training, the surgeons were shown how to remove as much of the cyst wall as possible to preserve functional bile ducts and cut non-functional bile ducts, examining all the surgical surfaces for bilio-cystic communications to decrease bile leakage. As a result, postoperative complications decreased from 15.0% to 9.0%.

Recurrences (i.e., cyst formation in the same place) may be related to the management of the residual cavity. For active echinococcal cysts, removing the residual capsule wall in its entirety is important, and avoidance of even minute spillage of cyst contents is a fundamental objective of primary hydatid surgery [28]. One aspect of our training was to teach surgeons how to protect the surgical area and how to use the rotary cutting machine to screw endocysts, minimizing the risk that protoscoleces spill and avoiding contamination of the operating area. 20% saline was used as an effective scolecidal agent. However, these steps were poorly performed in the 11 hospitals prior to training, which may have contributed to high recurrence rates. Albendazole may play a role in preventing recurrence after operation [29, 30]. According to expert consensus in China, the recommended dosage is 10–15 mg/kg/day, 3–7 days preoperatively and 3–12 months postoperatively for open cyst operations [18]. After the implementation, there was an increase in the number of doctors' recommendations for albendazole use before and after the operation. However, the preoperative prophylactic use of albendazole was still low (20.53%), which is something we need to continue to improve. Fortunately, after the implementation, there was a significant decrease in the number of doctors' recommendations for albendazole after radical surgery, indicating that the use of albendazole by doctors was becoming more and more standardized.

In our study, CE1, CE4, and CE5 patents also underwent surgery, which is not recommended by expert consensus [16]. We reviewed some of these cases: the surgically removed CE1 cysts were mainly large, superficial, or close to major vessels or the right kidney and adrenal gland. Some cases had no documented reasons recorded; however, it is possible that surgical treatment is embedded in traditional Chinese culture even though doctors suggest conservative treatment. In addition, for some symptomatic hydatid cysts, we still recommend surgical treatment.

This program improved knowledge and awareness of hydatid disease. In addition, preoperative CT evaluation, intraoperative technique, and postoperative drug use are likely to have had a positive impact on surgical outcomes. We believe that surgeons need to use the information provided by contrast enhanced CT to evaluate the overall morphology of hydatid cysts, the surrounding duct segments, and the incidence of complications to determine the optimal surgery to perform and increase its safety. Ultrasound does not provide as much information as CT for guiding surgery. Although it has been reported that MRI with a heavily T2-weighted series is preferable to CT [19], we still recommend contrast enhanced CT for preoperative evaluation due to its widespread availability and, locally, shorter waiting times and cost.

Unfortunately, it's very difficult to design a clinical study to find out the influencing factors of the surgical outcome of HCE, and we can't see that from this study. We can only infer from our experience for the influencing factors and conduct trainings.

In conclusion, surgery remains a major treatment modality for HCE, but its utility depends on the quality of the operation, improving operative safety where possible, and reducing postoperative recurrences and complications. We implemented an integrated HCE management plan with training that improved the knowledge, attitudes, and behavior of medical teams and decreased postoperative complications and recurrences. This integrated protocol is available, accessible, acceptable, adoptable, and affordable and can potentially be further adopted in other endemic areas.

## Supporting information

S1 Text A 12-item questionnaire for health care personnel before and after training to determine their knowledge about echinococcosis. (DOCX)

S2 Text CT training in this program, which includes CT imaging of the vessels and bile ducts surrounding hydatid cysts. (DOCX)

S3 Text Surgical training in this program, which includes the demonstration of the implementation surgery techniques. (DOCX)
