# Correction: An integrated surgical training program for hepatic cystic echinococcosis in Xinjiang of China

**DOI:** 10.1371/journal.pntd.0010551

**Published:** 2022-06-15

**Authors:** Hongwei Zhang, Jian Yang, Jiang Li, Jing Yang, Yunbao Yu, Guisheng Liu, Yongguo Zhang, Long Zhang, Wei Guo, Abuduwayiti, Hong Sun, Shuxia Guo, Xueling Chen, Xiangwei Wu, Shijie Zhang, Xinyu Peng

All figures were incorrectly reported in this article [1]. The figures were presented in the wrong order and were not matched with the corresponding figure legends. Corrected figures and corresponding figure legends are provided here; the titles and/or legends of some figures have been updated to relay more clearly the figure contents.

In addition, the authors provide the following updates to the Methods section, to provide details as to data collected and cases analyzed prior to program implementation.

The retrospective study (analysis of patients treated before implementation) analyzed data for surgical procedures conducted at Xinjiang Production and Construction Corps Hospital; Xinjiang Production and Construction Corps Fourth Division Hospital; Fifth Division Hospital; Sixth Division Hospital; Ninth Division Hospital; Tenth Division Hospital; Thirteenth Division Hospital; Hami Prefecture Central Hospital; Altay Region People’s Hospital; Usu City People’s Hospital; and Shawan County People’s Hospital.between January 2006 and January 2009, with 36 months follow-up. For analysis of outcomes after implementation of the program, data were collected on new HCE cases that were surgically treated at the same 11 above mentioned hospitals between January 2010 and January 2012, with a 36 month duration follow-up.

Inclusion criteria: (1) patients with hepatic cystic echinococcosis who are able to tolerate surgery and routinely undergo open abdomen surgical treatment;(2) the case data is complete, and no important data is missing.

Exclusion criteria: (1) patients with hepatic cystic echinococcosis who underwent emergency surgery (most of whom had hydatid complications, such as rupture);(2) patients who underwent surgery at other sites except for liver hydatid;(3) Laparoscopic treatment cases; (4) abnormal discharge patients.

Indications for surgery:

(1) Removal of large CE2-CE3b cysts with multiple daughter vesicles, (2) single liver cysts, situated superficially, that may rupture spontaneously or as a result of trauma, (3) cysts exerting pressure on adjacent vital organs or vessels.

Contraindications for surgery:

Surgery is contraindicated in patients to whom general contraindications for surgery apply, inactive asymptomatic cysts, difficult to access cysts, and very small cysts.

The baseline survey and the post-intervention survey of the knowledge of the medical staff used the questionnaire method. The case study uses the data collected from the cases, including: name, gender, age and other general information; Imaging diagnostic information (cyst location/ number/ size, etc.); Preoperative treatment; Operative methods, operation duration, blood loss, problems during operation, etc. Postoperative treatments, complications collection and judgment; The patients were followed up mainly to collect the patients’ liver and kidney function recovery status and to review the abdominal B ultrasound, whether it suggested recurrence and record the time.

The published Data Availability Statement is incorrect, the updated statement is provided here:

The primary data underlying results in this article cannot be made publicly available due to patient privacy concerns. Anonymized data are available upon request to: Hepatobiliary surgery department, the first affiliated hospital, school of medicine, Shihezi University, 107 north 2 road, Shihezi City, Xinjiang province, China 832008 (Email: zhw0108@163.com; pengxinyu2000@sina.com)

**Fig 1:**
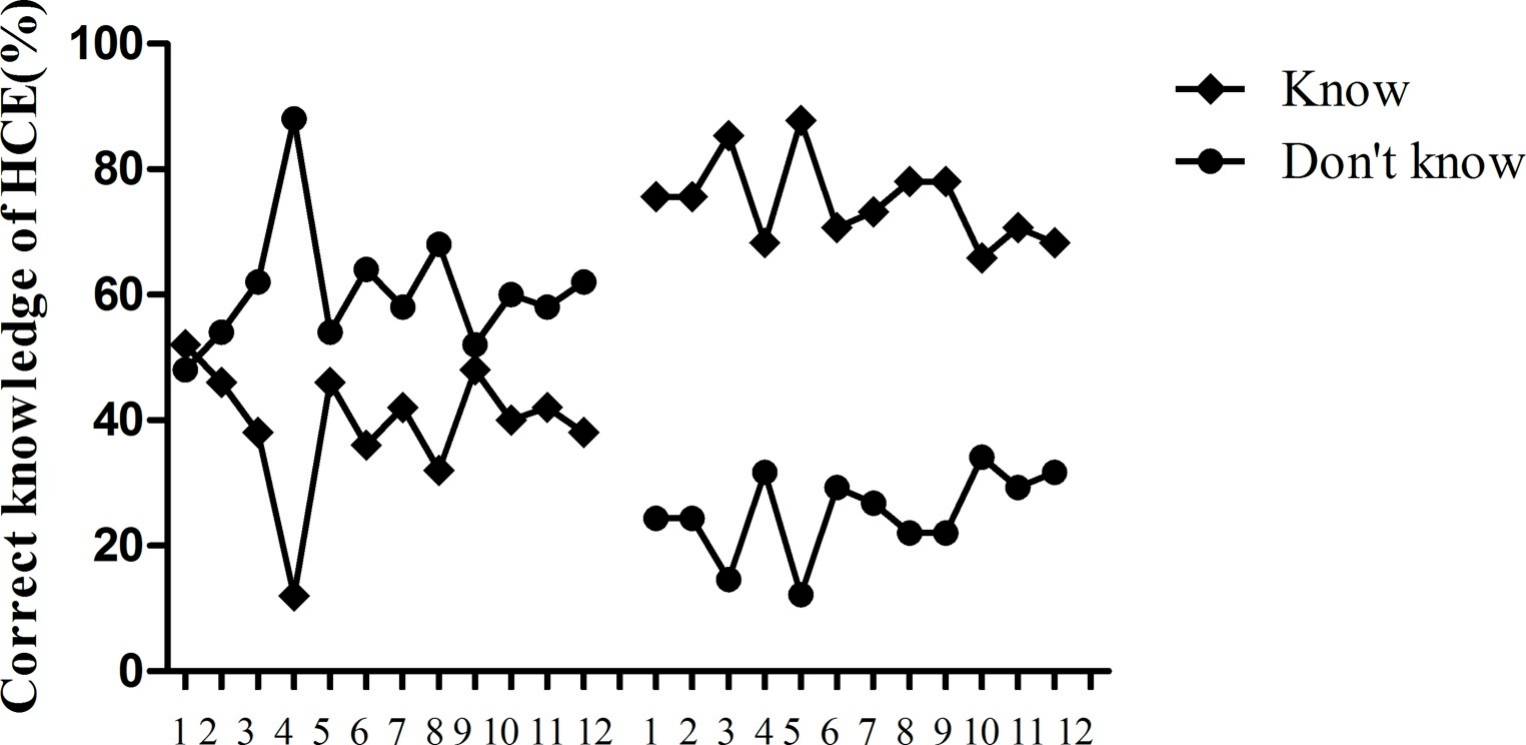
HCE knowledge of health care personnel before and after program implementation. Before program implementation, HCE knowledge of most of health care personnel was poor. After program implementation, knowledge increased. (p<0.05 by the chi-square test.) 1–12 denote the following questions: 1. Characteristics of echinococcosis; 2. The definitive host and intermediate host of cystic echinococcosis; 3. Most affected organs; 4. Drug of first choice; 5. Best radical surgery; 6. Intraoperative scolecidal agents and acting time; 7. Precautions in radical surgical approaches; 8. Sub-adventitial cystectomy in the surgical treatment of HCE; 9. Contrast enhanced CT application in HCE diagnosis and management; 10. Immunological diagnostic methods; 11. Processing methods of slaughtered livestock with hydatid cysts; 12. Hydatid specimen collection and storage.

**Fig 2:**
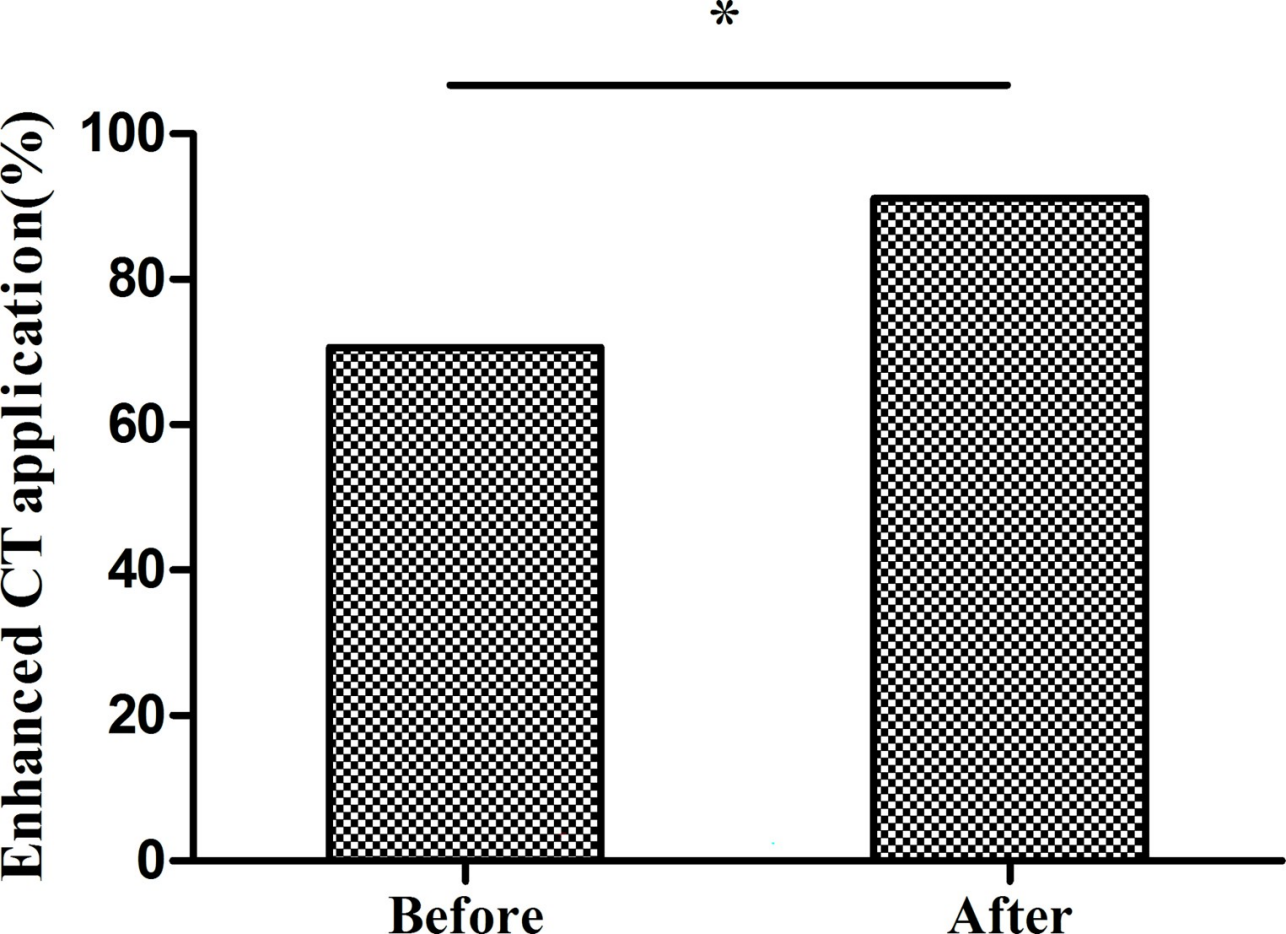
Contrast enhanced CT use before and after program implementation. The proportion of exams using enhanced CT increased from 70.7% to 91.1% after program implementation. (*p<0.001 by the chi-square test).

**Fig 3:**
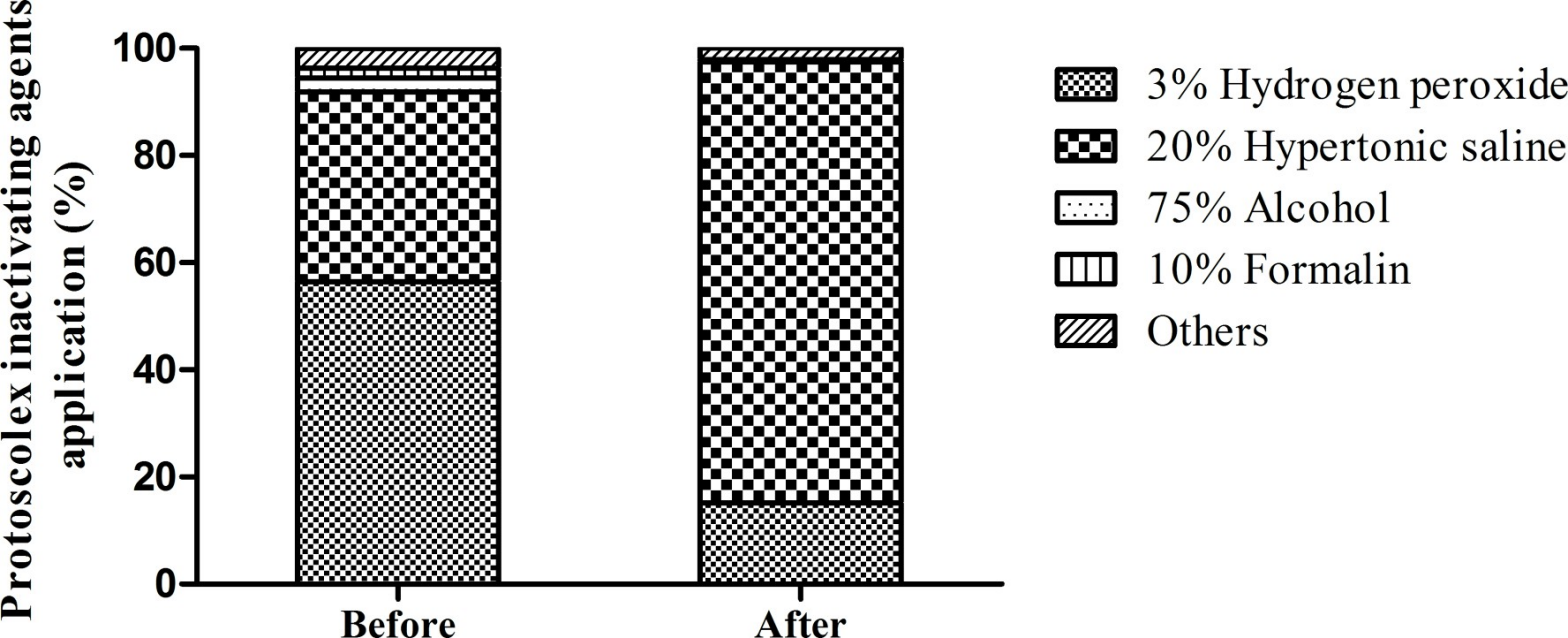
Use of scolecidal agents in open cyst operations before and after program implementation. Before program implementation, 3% hydrogen peroxide was used in 56.6% of cases and hypertonic saline in 35.4% of cases. After program implementation, hypertonic saline used increased to 82.3% and the application of 10% formalin decreased to 0%. The overall changes had significance of p<0.001 by the chi-square test.

**Fig 4:**
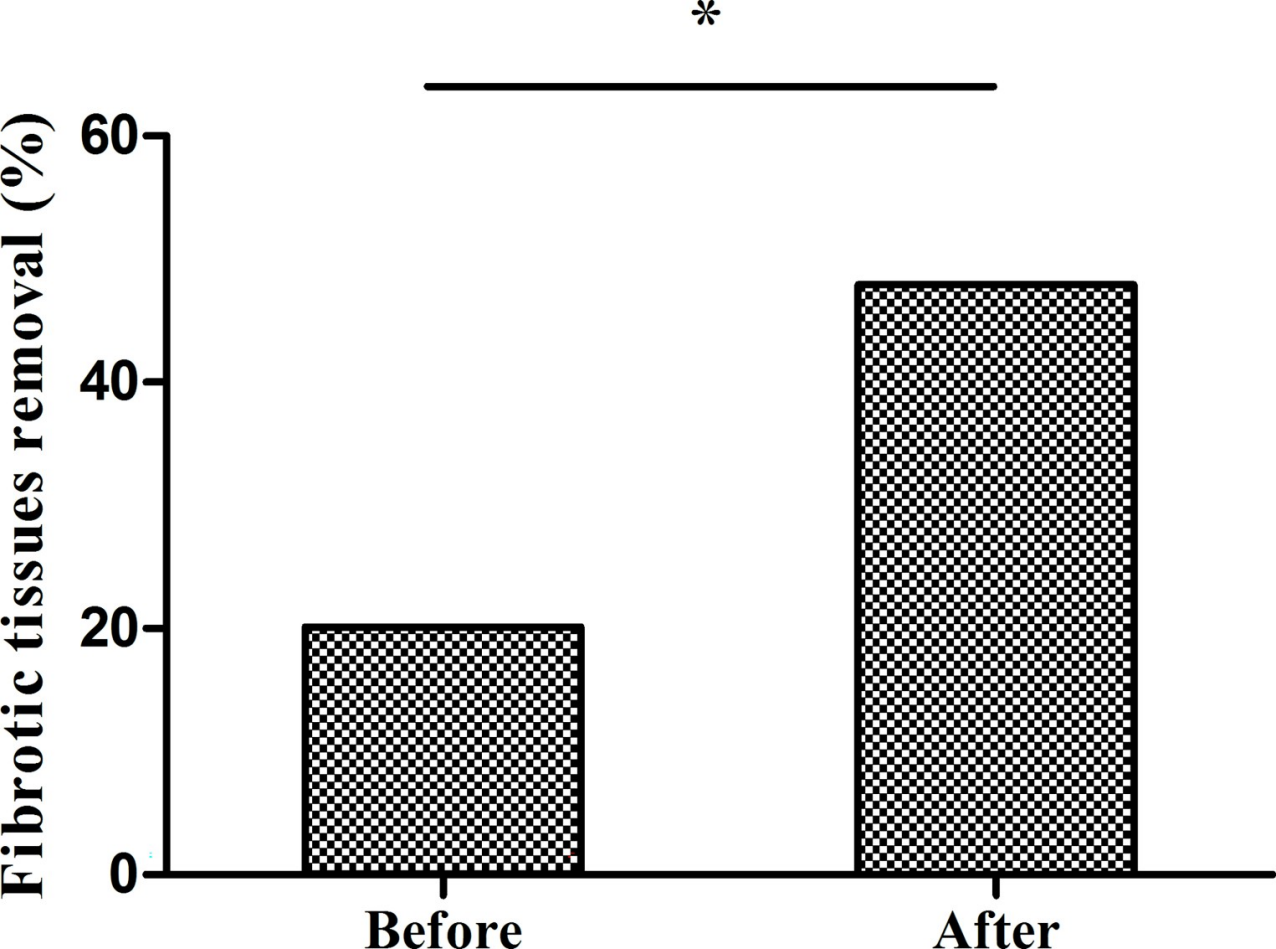
Handling of fibrotic and necrotic tissues during open surgery before and after program implementation. Before program implementation, only 20.1% of surgeries involved removing necrotic and fibrotic tissue. After program, implementation, the proportion increased to 47.9%. (*p<0.001 by the chi-square test).

**Fig 5:**
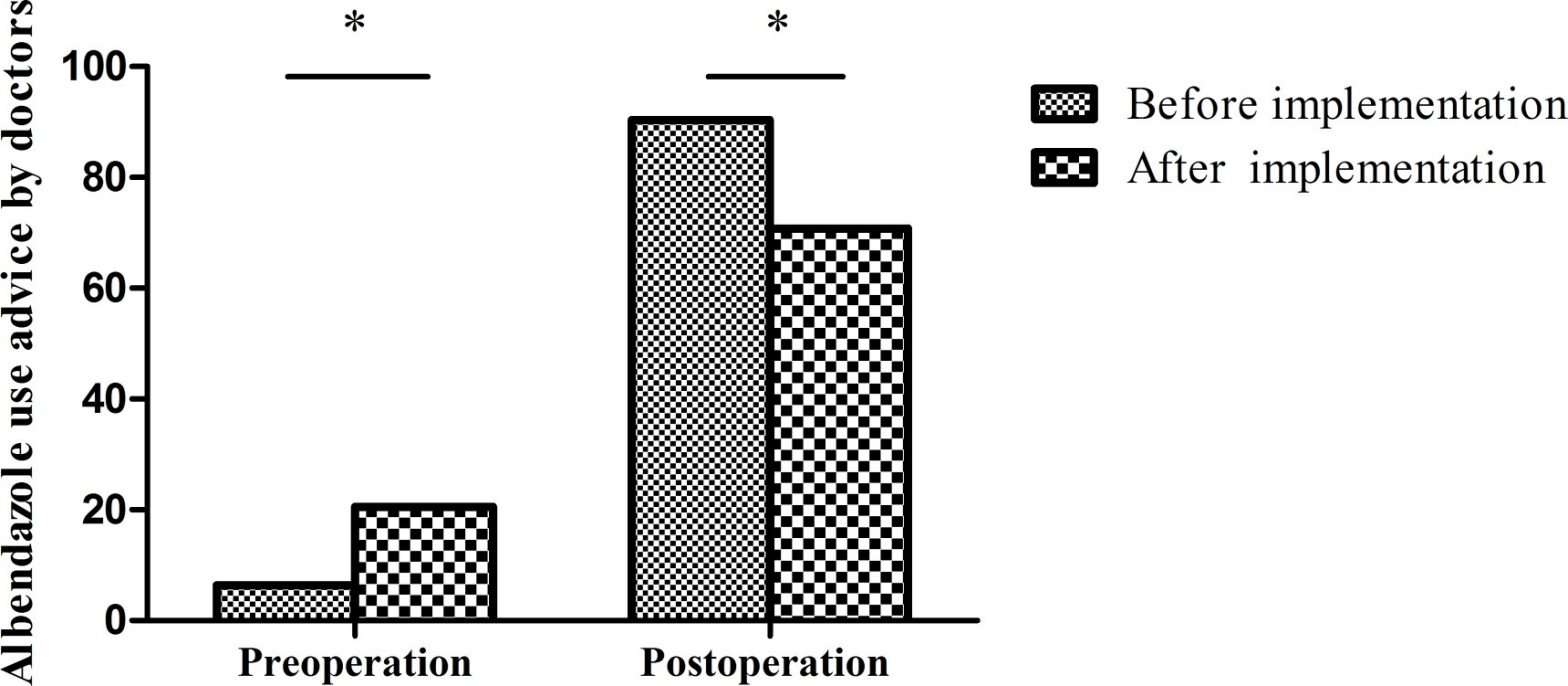
Albendazole use before and after program implementation. Before program implementation, preoperative use of albendazole was low. After program implementation, preoperative use of albendazole use increased from 6.4% to 20.5%. (*p<0.001 by the chi-square test).

**Fig 6:**
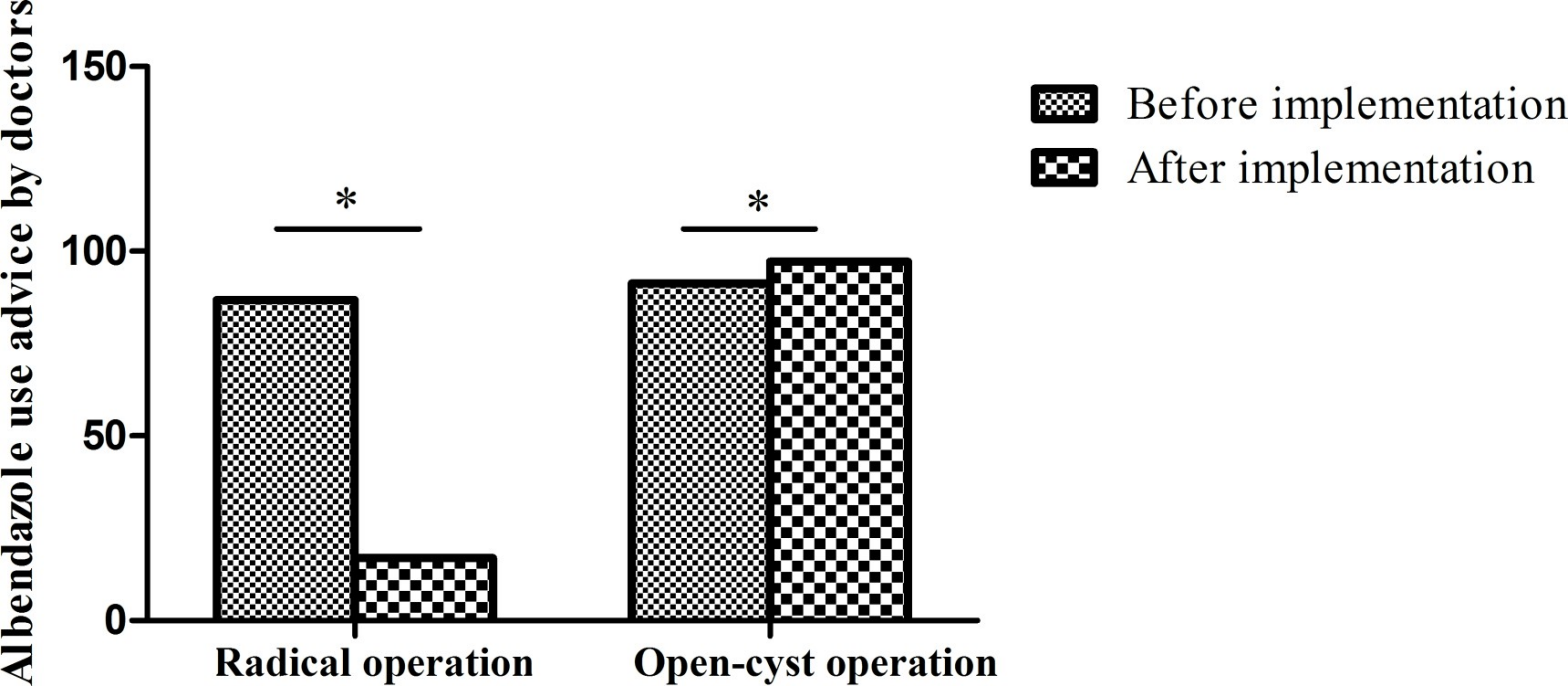
Postoperative discharge guidance for abendazole use following radical and open-cyst operation. Based on doctors’ discharge advice, the recommendation to use abendazole following open-cyst operations increased after implementation, while recommendations to use abendazole after radical operations significantly decreased. (*p<0.001 by the chi-square test).

**Fig 7:**
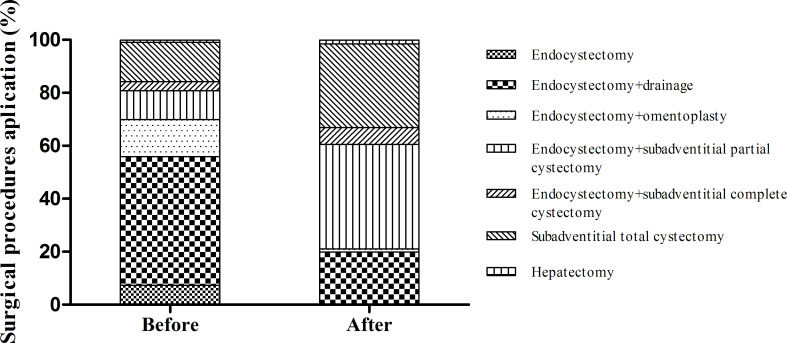
The proportion of patients undergoing radical surgery was reduced after program implementation. Endocystectomy and its derivatives (drainage; omentoplasty) decreased after program implementation; especially the sole use of endocystectomy (0%). Radical surgery, especially subadventitial (total and complete) cystectomy, increased. (*p<0.001 by the chi-square test).

**Fig 8:**
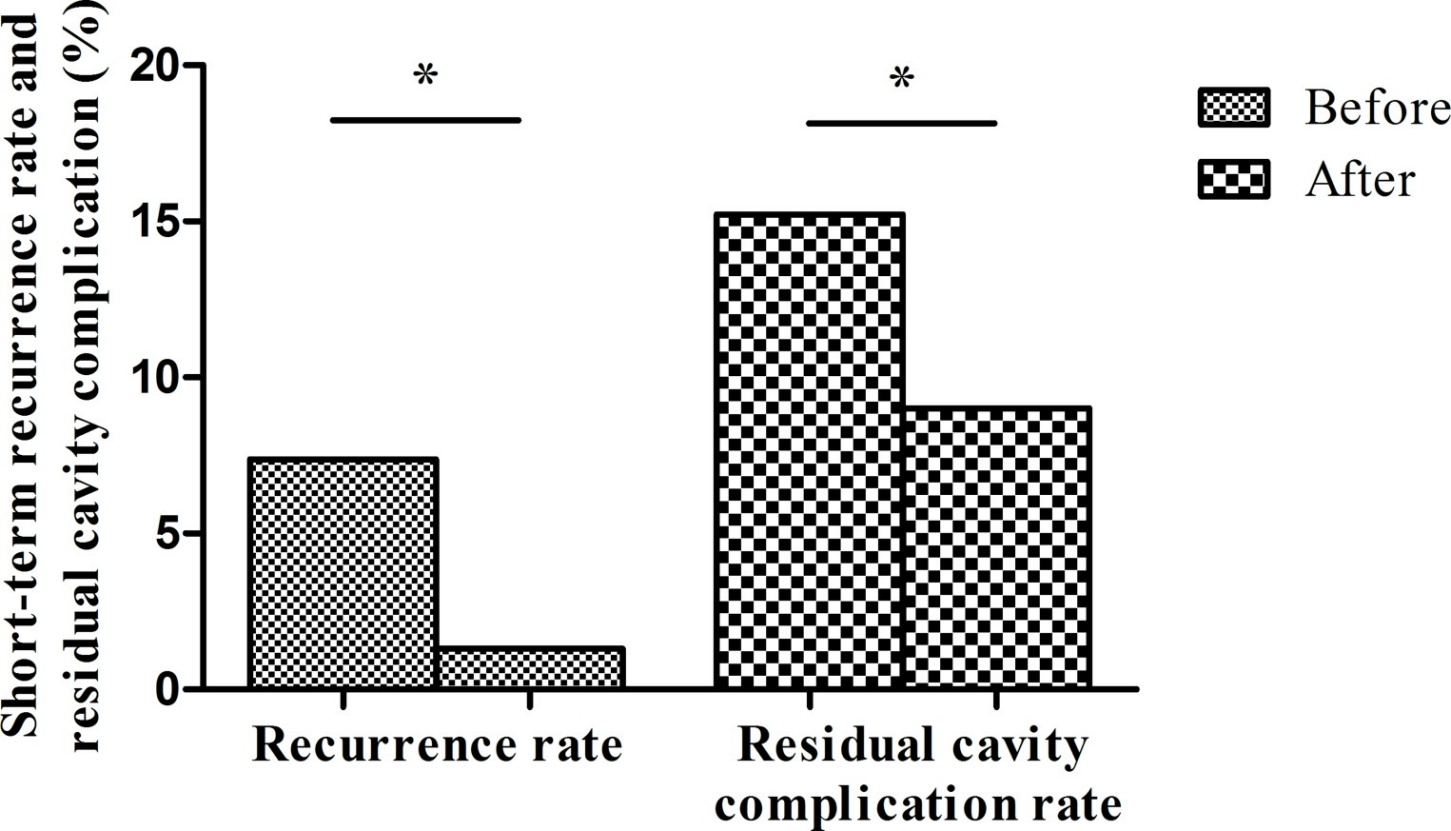
Decreases in short-term overall postoperative recurrences and residual cavity complications after program implementation. Recurrences decreased from 7.4% to 1.3%, while residual cavity complications decreased from 15.2% to 9.0%. (*p<0.001 by the chi-square test).

**Fig 9:**
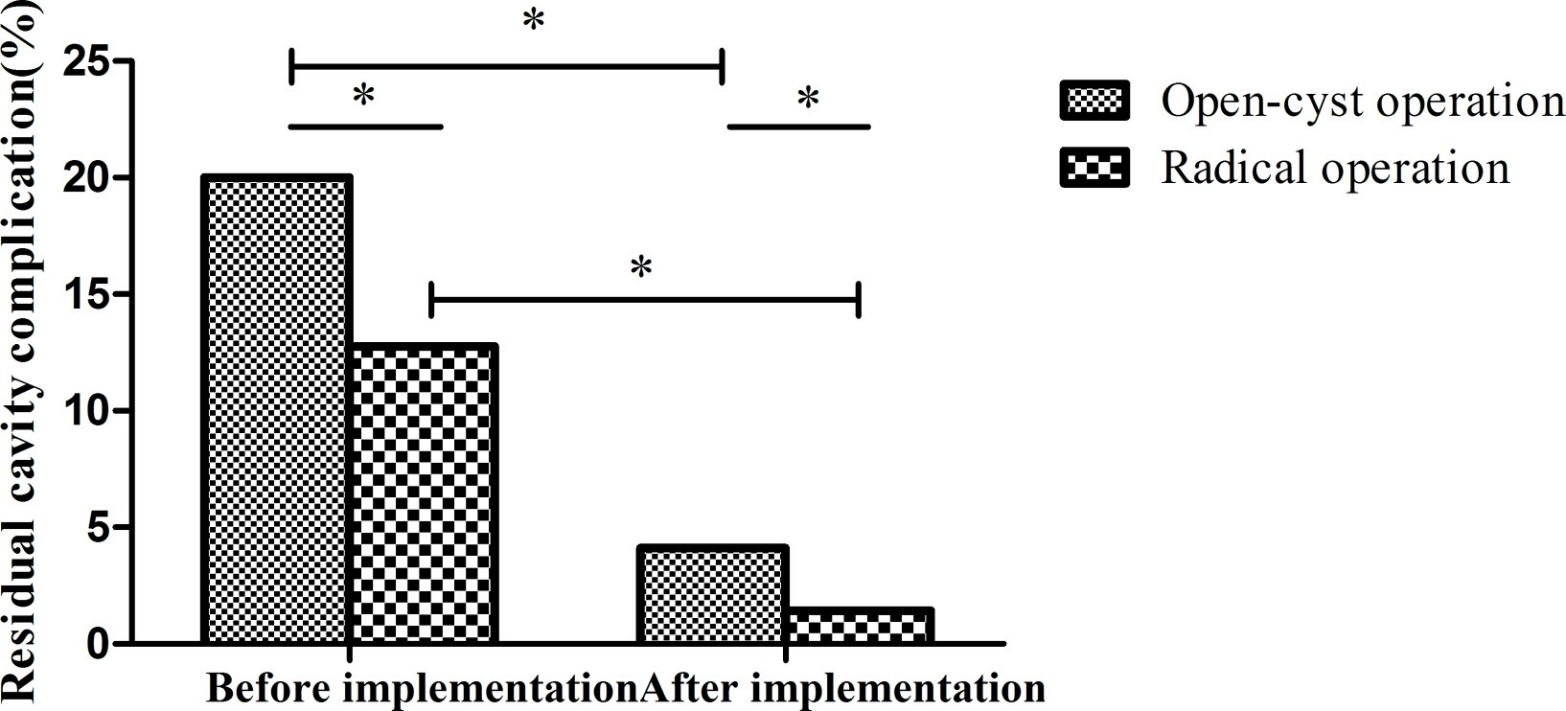
Complication rates following open-cyst versus radical operations. After program implementation, the residual cavity complications of the open- and closed-cyst operation decreased compared with that before the implementation, and the differences were statistically significant. (*p<0.001 by the chi-square test).
