# Supplementary material for: An integrated surgical training program for hepatic cystic echinococcosis in Xinjiang of China
Source: PLoS Negl Trop Dis. 2020 Mar 12;14(3):e0008023. doi: 10.1371/journal.pntd.0008023 (PMC7093013; doi:10.1371/journal.pntd.0008023)
Supplement: S1 Text — (DOCX) [file pntd.0008023.s001.docx]

**Supplemental Materials**

**HCE Knowledge Training**

The content of knowledge training was primarily about the life cycle, transmission, pathology, symptoms, diagnosis, treatment, and other aspects of cystic hydatidosis. Based on international expert consensus and Chinese guidelines, the relevant medical personnel in 11 hospitals were trained through training courses, study manuals, videos, and other methods. Surgeons, ward nurses, operating room nurses, anesthesiologists, laboratory physicians, imaging physicians, and other relevant personnel received training.

We designed an examination paper (below) to assess staff knowledge on hydatid disease. The examination paper was in multiple choice format based on international expert consensus and national guidelines. We marked each question as correct or incorrect to determine the degree of mastery of each item.

**HCE Knowledge Investigation for Medical Staff**

| Hospital Name: |  | Education degree: |  |
| --- | --- | --- | --- |
| Staff Name: |  | Age: |  |
| Working unit: |  | Working duration: |  |
| Title: |  |  |  |

**1. What is *NOT* the character of hepatic cystic echinococcosis (HCE)? (D)**

A. Local epidemics

B. Natural epidemics

C. Zoonosis

D. Blood transmission

**2. Which is the most common definitive host and intermediate host of cystic echinococcosis (CE) in grazing areas? (D)**

A. Dogs, humans

B. Foxes, humans

C. Sheep, humans

D. Dogs, sheep

**3. Which two organs are most affected by CE? (A)**

A. Liver and lung

B. Lung and brain

C. Liver and peritoneal cavity

D. Lung and peritoneal cavity

**4. Which is the first line drug for the treatment of HCE? (B)**

A. Antibiotics

B. Albendazole

C. Artemisinin

D. Quinolone

**5. Which of the following can be considered a radical procedure for HCE? (C)**

A. Percutaneous puncture

B. Radiofrequency [ablation](javascript:;) ([RFA](javascript:;))

C. Total cystectomy

D. Endocystectomy

**6. What is the recommended intraoperative scolecidal agent and how long should it be applied for? (A)**

A. 20% hypertonic saline for at least 15 min

B. Albendazole for at least 15 min

C. Hydrogen peroxide for at least 15 min

D. 10% formaldehyde for at least 15 min

**7. Which is *NOT* true with respect to the perioperative management of radical surgical HCE cases? (B)**

A. CT evaluation of the hydatid cysts before surgery

B. B ultrasound is sufficient for surgical evaluation

C. Scolecidal agent preparation before surgery

D. Albendazole 3-7 days before surgery

**8. Which of the following statements is *false* with respect to sub-adventitial cystectomy in the surgical treatment of HCE? (B)**

A. It is recommended as a choice for surgical treatment by WHO experts

B. Remove the cyst through the healthy liver tissue

C. Can be performed in a closed total way

D. Can be performed in an open-cyst way

**9. Which of the following statements is true with respect to enhanced CT for the diagnosis and guidance of treatment of HCE? (B)**

A. Every patient needs one to diagnose HCE

B. Should be performed in surgical patients

C. Shows less clear pericystic structures than B ultrasound

D. Only for symptomatic patients

10. **Which is *NOT* an immunological diagnostic method? (D)**

A. Cassoni test

B. Indirect agglutination test

C. Complement binding test

D. AFP

**11. Which is the right processing method for slaughtered livestock organs with hydatid cysts? (C)**

A. Can't be eaten, just throw away

B. Can be given to dogs

C. Deep bury the organs

D. The livestock can’t be eaten and throw away with the infected organ

**12. Which is the right way to take and store hydatid specimens after surgery? (A)**

A. Fresh tissue should be taken in 15 minutes and stored in a -80℃ ultra-low temperature freezer

B. Fresh tissue should be taken the next day and stored in a normal freezer

C. Fresh tissue should be taken in 15 minutes and stored in H_2_O_2_

D. Take and use the spacemen freely without the informed consent from patients
