# Supplementary material for: An integrated surgical training program for hepatic cystic echinococcosis in Xinjiang of China
Source: PLoS Negl Trop Dis. 2020 Mar 12;14(3):e0008023. doi: 10.1371/journal.pntd.0008023 (PMC7093013; doi:10.1371/journal.pntd.0008023)
Supplement: S2 Text — (DOCX) [file pntd.0008023.s002.docx]

**CT training including:**

(1) Training course: the main content is to teach the importance of CT imaging diagnosis and perioperative evaluation, to evaluate the overall growth of hydatid cyst, to determine the degree of difficulty of surgery, and the relationship with surrounding blood vessels and bile ducts;

(2) Establishment of remote consultation system to provide guidance for 11 hospitals to read films remotely and help them diagnose and develop treatment plans.

**
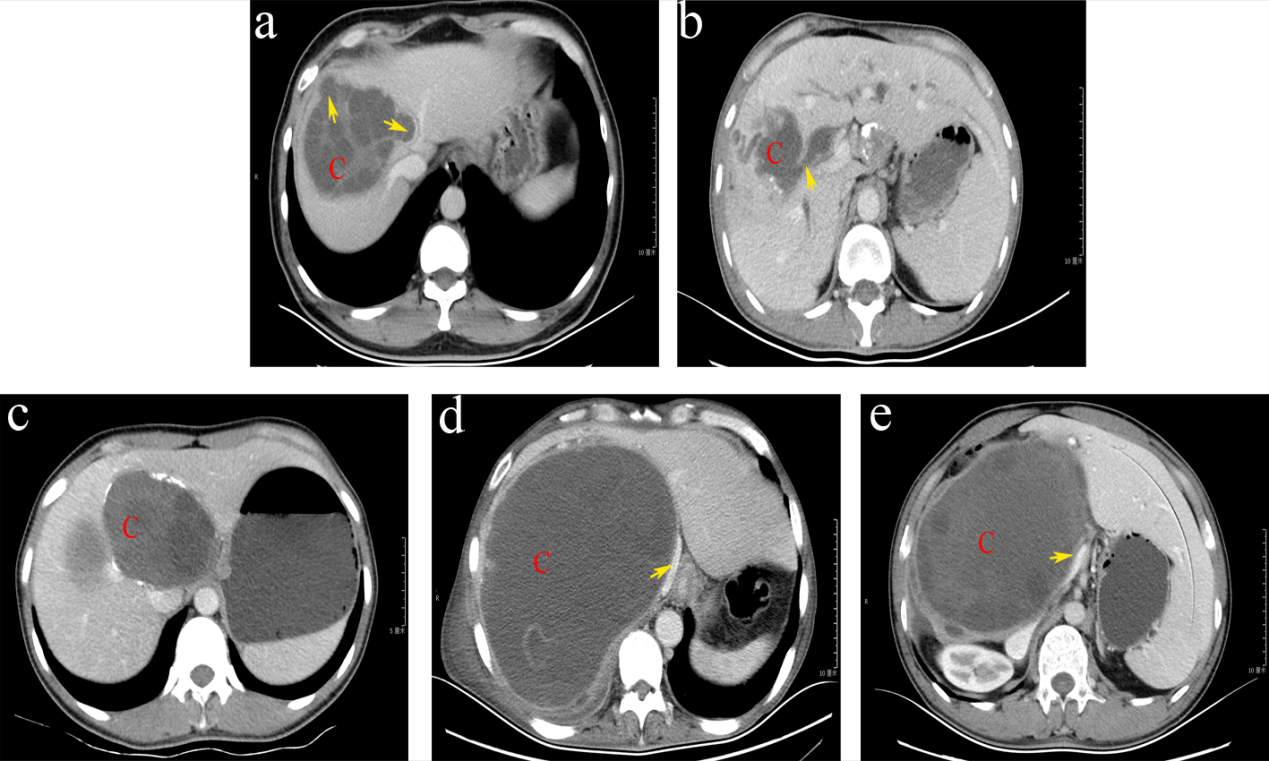
**

**S1 Figure. CT imaging of the vessels and bile ducts surrounding hydatid cysts.** a. Intrahepatic breakthrough growth of a CE2 cyst (yellow arrow); b. Cystobiliary communication (yellow arrow); c. A CE3b cyst adjacent to the first portal hilar branch; d. A CE2 cyst adjacent to the inferior vena cava (yellow arrow); e. CE3b cyst adjacent to the portal vein (yellow arrow). Abbreviation: C, hydatid cyst.
