# Supplementary material for: An integrated surgical training program for hepatic cystic echinococcosis in Xinjiang of China
Source: PLoS Negl Trop Dis. 2020 Mar 12;14(3):e0008023. doi: 10.1371/journal.pntd.0008023 (PMC7093013; doi:10.1371/journal.pntd.0008023)
Supplement: S3 Text — (DOCX) [file pntd.0008023.s003.docx]

**The implementation of surgical training include:**

(1) Training course: mainly teach the pathological knowledge, surgical anatomy and operation video broadcasting of echinococcosis.

(2) On-site guidance: 2-3 surgical experts designated by the Ministry of Health of the People's Republic of China conducted on-site surgical demonstration in 11 selected hospitals to further explain the knowledge and operation steps in the teaching and training classes.

(3) Assessment: The key operation steps were assessed by 2-3 surgical experts designated by the Ministry of Health of the People's Republic of China., and then gave feedback for further improvement. The operation assessment conducted twice to ensure the selected surgeons mastered the operation method and key surgical techniques.

**Surgical procedures description.**

**(1) Subadventitial close total cystectomy:** Following general anaesthesia, a subcostal or upper midline “┛” incision was made. Liver was fully mobilized to make sure the cyst was fully exposed in the surgical field and portal triads slung in preparation for Pringle maneuver when needed. The virtue space between cyst and liver parenchyma was identified by retracting the liver to the opposite side of cyst wall using PMOD ( Peng’s multifunctional operative dissector, which combined an electrotome with an aspirator, Figure 1 black arrow) followed by electro-coagulation. The correct space was usually indicated when little bleeding encountered during dissection, and the surface of liver parenchyma covered by a membranous layer(Figure 1 yellow arrow). Gradually, the cyst was separated along this space from the liver parenchyma in a way like “peel an orange” by a series of coagulation, pushing, scraping, suction”. During this process, the non functional ducts were ligated and divided, and the functional ducts (bile ducts or vessels) preserved. The cyst was resected totally without disruption of its integrity. If the cyst is too large or complex, Subadventitial open total/partial cystectomy is

considered.


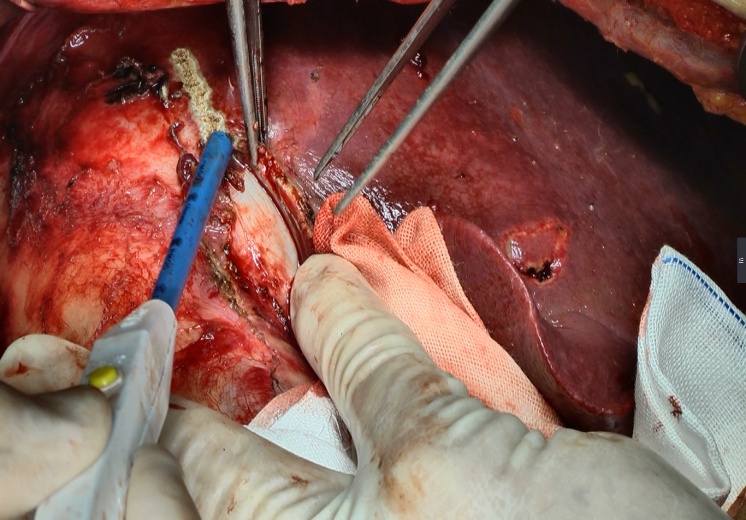


Healthy liver

Hydatid cyst

Fig. 1 Demonstration of Subadventitial close total cystectomy. black arrow: PMOD, yellow arrow: the right space between the healthy liver parenchyma and the cyst.

**(2) Subadventitial open total/partial cystectomy:** Following general anaesthesia, a subcostal or upper mid line “┛”incision was made. Before decompression of cyst, 20% saline(Figure 2), and rotary cutting (Figure 3) and suction system(Figure 4) were prepared. After the protection of the 20% saline swab covered by surgical film, a small incision and simultaneous suction was made by the electrocautery PMOD to cyst was punctured(Figure 4, 5), and incision enlarged for insertion of  rotary cutting system, to rotary cut and suck out the contents via this system to avoid intra abdominal contamination, which may possibly result in peritoneal implantation and anaphylactic shock. A close inspection for cystobiliary communication should be performed after the content of the cyst is removed which should be dealt with adequately with suturing followed instillation of 20% saline to the residual cavity for 15 min to inactivate the protoscoleces, and then suck out the saline. Remove the protection and start to find the space between the cyst and liver parenchyma as mentioned above. The remnant cyst is removed as much as possible along the space and ducts as mentioned above. The fibrous tissue on the remnant part of the pericyst needs to be cleared as much as possible (Figure 6). Exploration of the remnant cyst wall for cystobiliary communication must be completed prior to finishing bearing in mind that several communications may exist.


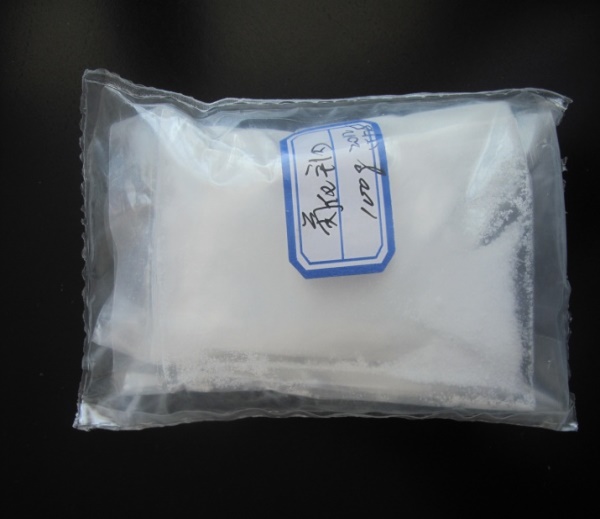

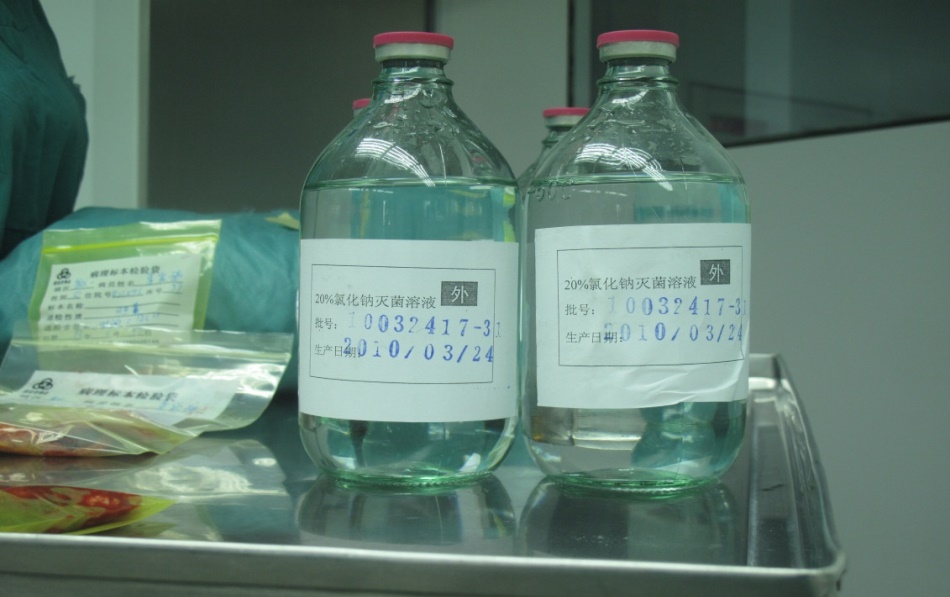


b

a

Fig. 2 a. package of sterile Nacl; b. 20% saline solution. We made these products and sent to the 11 hospitals for free use during the program.


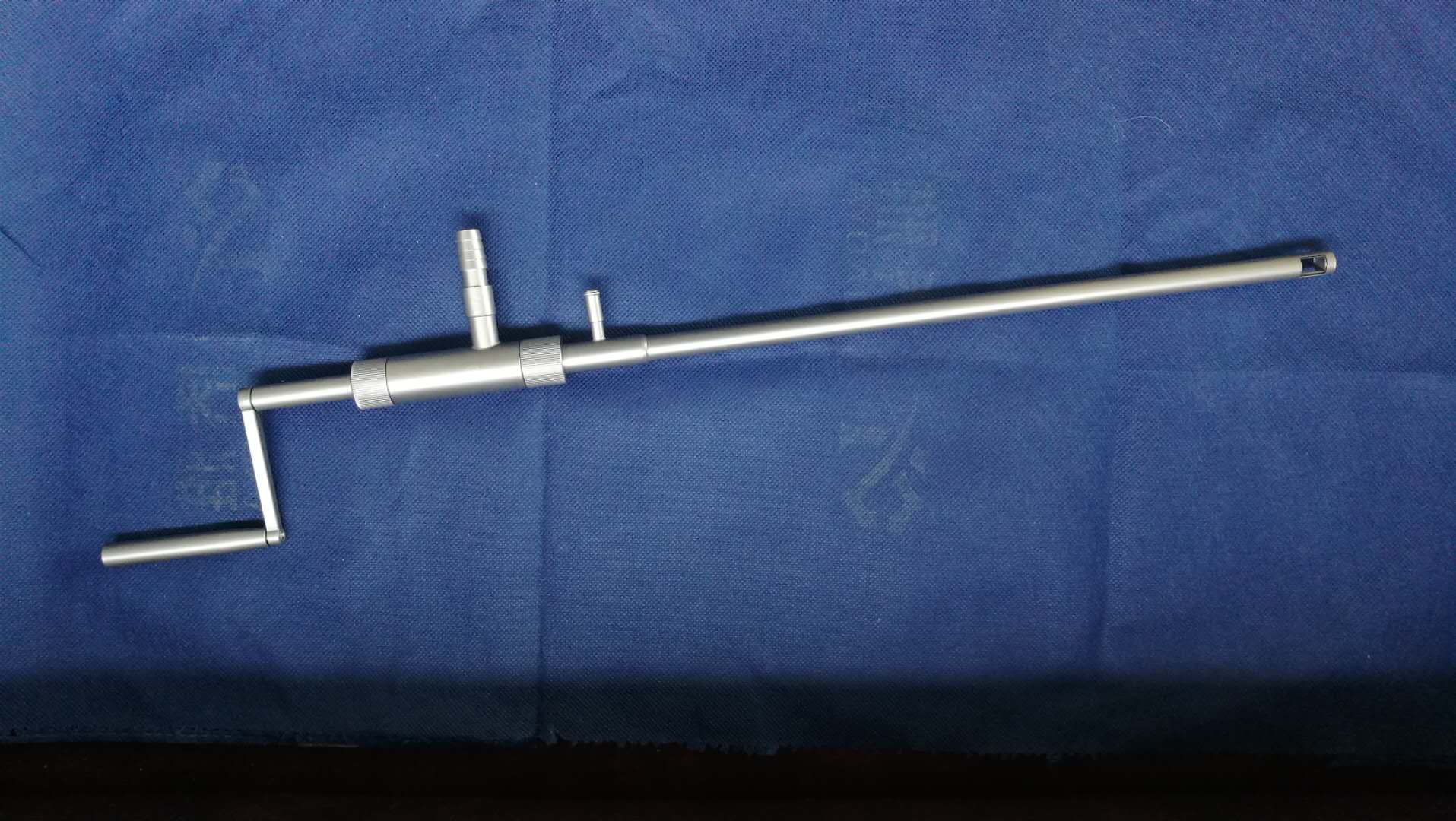


Fig. 3 The third generation of rotary cutting system (our patent product for removal of the content of the hydatid cyst). white arrow: [rocking](javascript:;) [handle](javascript:;); yellow arrow: connection to suction; red arrow: connection to normal saline; green arrow: rotary cutting blade.


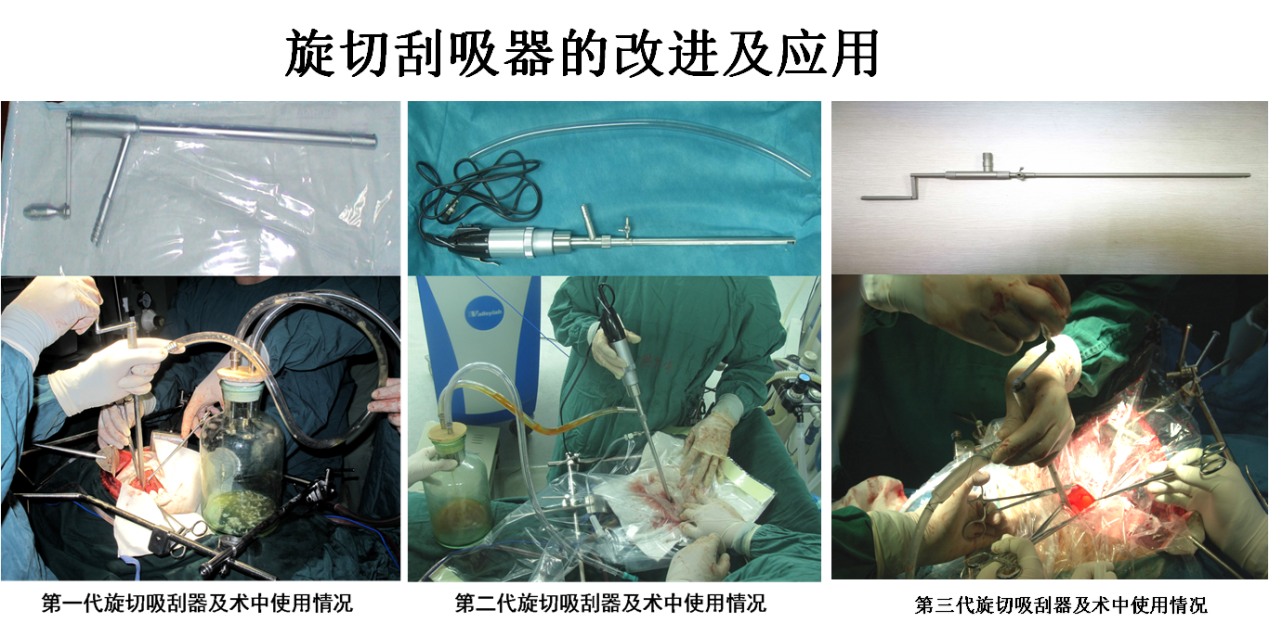
Fig. 4 the demonstration of the one- third generation of the rotary cutting system for removal of the content. A. the first generation; b. the second generation. C. the third generation. Yellow arrow: suction system.


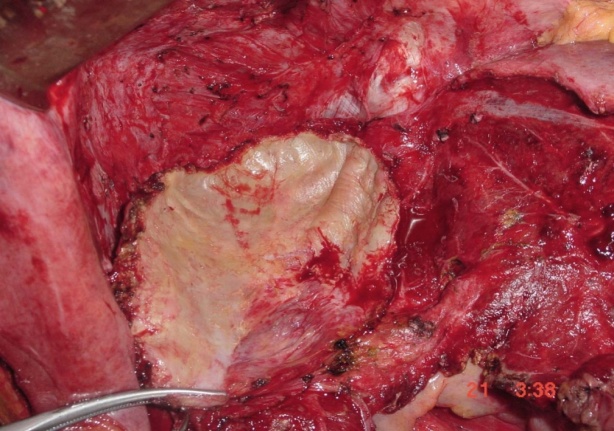

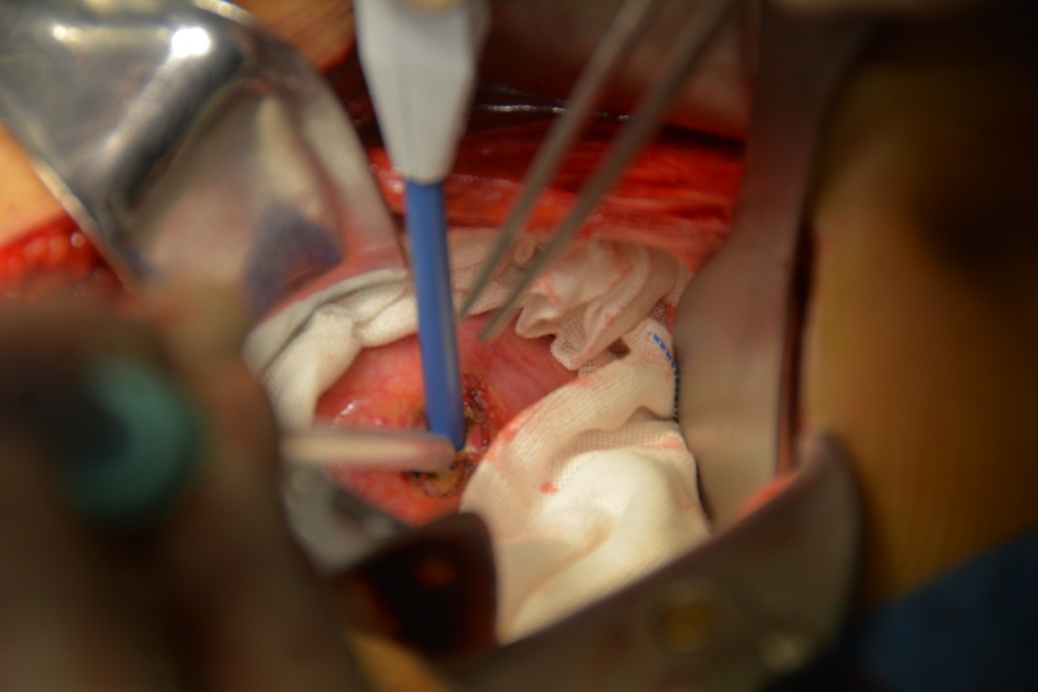


b

a

Fig. 5 The demonstration of Subadventitial open partial cystectomy. a PMOD was used to make a small incision on the hydatid cyst, where surrounded by 20% saline swabs (red arrow), allowing only the head of the PMOD to enter(white arrow), while assisting a second suction device to prevent overflow of the fluid. black arrow: PMOD; b. the remnant cystic wall.


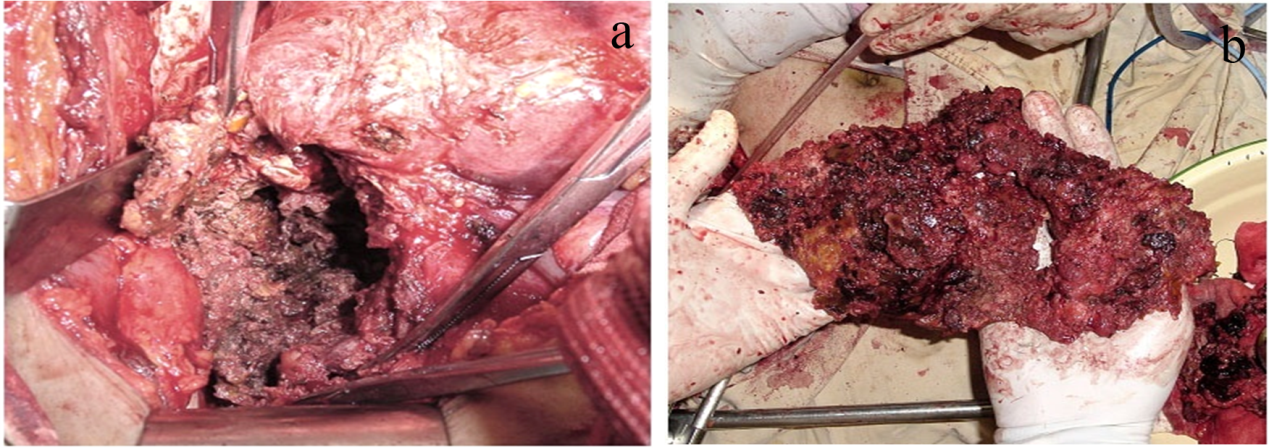


Fig. 6 Fibrotic tissue in hydatid cysts. a. The residual cavity after endocystectomy; b. A cystic wall removed as completely as possible.
